# Valorization of ductile cast iron solid waste as a high-performance adsorbent for crystal violet removal: characterization, optimization, and mechanistic insights

**DOI:** 10.1039/d6ra02129h

**Published:** 2026-05-11

**Authors:** Ibrahim M. Ibrahim, A. M. Turky, Nasser Y. Mostafa, Mai H. Roushdy

**Affiliations:** a Chemical Engineering Department, Faculty of Engineering, The British University in Egypt El-Shorouk City 11837 – Cairo Egypt mai.hassan@bue.edu.eg; b Department of Chemistry, Faculty of Science, Suez Canal University Ismailia 41522 Egypt

## Abstract

Industrial textile wastewater containing synthetic dyes cause serious environmental and health risk, whereas ductile cast iron (DCI) foundries generate over 500 000 tons of waste annually. This study utilizes DCI solid waste as an adsorbent to remove the crystal violet (CV) dye from wastewater. Techniques (XRF, XRD, BET, SEM-EDX, FTIR, TGA-DTG, zeta potential) proved that the waste contains 88.0 wt% periclase (MgO) with nanoscale, high surface area, and abundant surface hydroxyl groups. Response surface methodology showed that the most significant parameters were the adsorbent dose and time contact. The optimal conditions give 93.7% removal efficiency at initial concentration: 38.7 mg L^−1^, adsorbent dose: 6.2 g L^−1^, shaking rate: 150 rpm, and contact time: 30 min. The isotherm model was the Freundlich model suggesting surface heterogeneity with dispersed binding energies; multilayer coverage supports this, but the Freundlich fit by itself cannot establish it. The maximum physisorption capacity was 116.85 mg g^−1^, and the mean free energy, *E* = 3.34 kJ mol^−1^. Kinetic study demonstrated that the reaction follows pseudo-first-order kinetics (*k*_1_ = 0.1654 min^−1^) and showed three diffusion phases: the external film diffusion (0–30 min), the intraparticle diffusion (30–120 min), and the equilibration phase (>120 min). The thermodynamic investigation showed that the adsorption is an endothermic process (Δ*H*° = +22.15 kJ mol^−1^), accompanied by a positive entropy change (Δ*S*° = +85.3 J mol^−1^ K^−1^) and a negative Gibbs free energy change (Δ*G*° = −2.94 to −5.68 kJ mol^−1^), which means spontaneous, entropy-driven physisorption. Post-adsorption XRD showed that MgO was hydroxylated to Mg(OH)_2_. The pH optimization revealed maximum removal at pH 7–9. The regeneration technique employing acid and thermal methods yielded a desorption efficiency rate of 95.4%, a cumulative adsorption capacity recovery rate of 78.5% following 15 cycles, and magnesium release lower than all permissible standards (USEPA, WHO, Egyptian Law 4/1994). The initial techno-economic analysis yields a unit treatment cost of approximately $1.09 m^−3^ for a hypothetical 1000 m^3^ d^−1^ plant; nevertheless, further confirmation based on scale up and continuous flow operation is essential prior to actual commercialization. This study proves that DCI solid waste is not only economically feasible but also environmentally adsorbent in the context of a circular economy.

## Abstract 2

Ductile cast iron waste efficiently remove crystal violet *via* electrostatic attraction and surface complexation, achieving 93.7% removal, high capacity, fast kinetics, and cost-effective regeneration for sustainable wastewater treatment.

## Graphical abstract

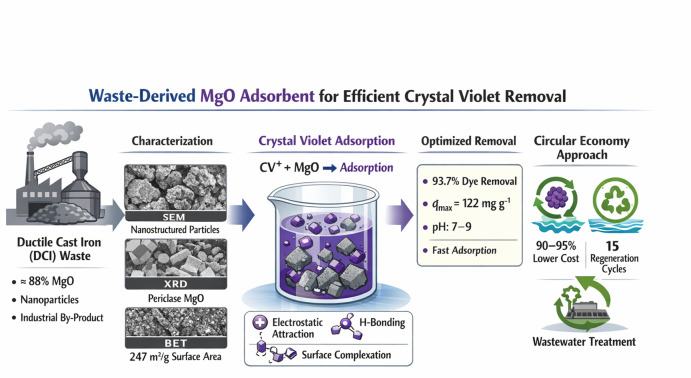

## 1 Introduction

Over 700 000 tons of synthetic dyes, roughly more than 10 000 commercially available dyes, are released into aquatic ecosystems each year, making industrial effluent containing synthetic dyes one of the world's most difficult environmental issues.^1,2^ Approximately 60–70% of the world's dye production is consumed by the textile sector alone, and 10–25% of it is believed to be discharged untreated into water bodies due to ineffective dyeing techniques and insufficient treatment facilities.^3,4^ The main synthetic dyes are divided according to their chromophore structures azo, anthraquinone, triphenylmethane, reactive, and vat (in fact, the cationic triphenylmethane dyes are the major environmental concern because these dyes are persistent, toxic, and are not easily degraded by the common treatment methods).^5–7^ The triphenylmethane cationic dye crystal violet is widely used in textile dyeing, paper printing, leather treatment, biological staining, and medicinal compositions.^8^ Acute toxicity to aquatic creatures, probable carcinogenicity, mutagenicity, developmental toxicity, and mitochondrial dysfunction in mammalian cells are just a few of the serious environmental and health risks associated with CV, despite its commercial significance.^2,9^

Because of its intricate aromatic structure with three dimethylamino groups that provide remarkable durability against biological degradation, photolytic breakdown, and traditional oxidation processes, CV is recalcitrant.^4,10^ Because of these structural features, CV remains resistant to the common treatment methods, all of which have considerable drawbacks in terms of effective and cost-efficient removal. Activated sludge processes, chemical coagulation–flocculation, membrane separation, and advanced oxidation are examples of conventional wastewater treatment techniques that show little effectiveness against CV and have high initial and ongoing expenditures.^11,12^ Microorganisms are highly sensitive to the toxicity of dyes, which is the main reason why biological treatment can only achieve a color removal efficiency of 30–50%,^13^ while membrane technologies are susceptible to fouling which leads to the need for costly membrane replacement.^14^ Although they are very efficient, advanced oxidation techniques consume more energy and produce dangerous chemical by-products that may pose a risk to human health.^11,15^

On the other hand, adsorption has become the leading method, technically and economically, for dye removal from wastewater. It offers a straightforward way of working along with excellent removal efficiency. Among the advantages are high removal efficiency (>90%), easy operation, low sludge production, no harmful by-products, and adsorbent regeneration potential. Adsorption has thus become the most promising technique for dye removal.^16,17^ However, activated carbon is still the industry standard adsorbent with removal efficiencies of more than 95% for various dyes,^18^ Extensive industrial use of activated carbon is, however, limited by high production costs ($1500 3000 per ton), energy-intensive regeneration requirements (800 1000 °C), and gradual capacity loss during regeneration cycles.^19^

Such limitations have led to a great deal of research on alternative adsorbent materials, most notably cheap precursors from mineral wastes, industrial leftovers, and agricultural residues.^20,21^ These are examples of agricultural waste-based adsorbents that are cheap but have low adsorption capacities (typically 20 80 mg g^−1^), long times to reach equilibrium (2 6 hours), and significant batch-to-batch variability: rice husk, coconut shell, and sugarcane bagasse.^22,23^ Fly ash, red mud, and steel slag are examples of industrial byproducts that have demonstrated potential but frequently require significant chemical or thermal alteration to improve performance, offsetting their economic advantages.^24,25^

Magnesium oxide (MgO) is one of the mineral-based materials that has been studied as a low-cost adsorbent alternative and has gained a lot of attention because of its outstanding physicochemical properties. Due to its special physicochemical characteristics, such as high surface basicity (p*K*a = 12.4), remarkable thermal stability (melting point 2852 °C), biocompatibility, and environmental benignity, magnesium oxide (MgO) has drawn a lot of interest as an adsorbent.^26,27^ Heavy metals (Pb^2+^, Cd^2+^, Cr^2+^), organic dyes, pharmaceutical residues, and fluoride are among the contaminants that MgO-based materials effectively remove.^28–30^ Electrostatic attraction between positively charged surfaces and anionic pollutants, surface complexation with metal cations, hydrogen bonding with organic molecules, and precipitation of insoluble chemicals are some of the processes involved in the adsorption mechanism.^31–33^

However, high-temperature calcination of magnesium-containing minerals (dolomite, magnesite) or precipitation from brine or seawater, followed by thermal breakdown are necessary for commercial MgO production.^27^ Large-scale wastewater treatment applications are not economically viable due to the $400–1200 per ton manufacturing costs associated with these energy-intensive procedures (usually 700–1000 °C to obtain high surface area).^34,35^ Furthermore, the high energy consumption (3–5 GJ per ton MgO) results in high carbon emissions (0.6–1.2 tons CO_2_ per ton MgO), which goes against sustainability goals.^36,37^

A vital engineering material, ductile cast iron (also known as nodular iron or spheroidal graphite iron) combines the castability and machinability of gray iron with mechanical qualities that are comparable to steel (tensile strength 400–800 MPa, elongation 2–18%).^38,39^ Over 27 million tons are produced worldwide each year for the automotive (40%), mechanical (25%), pipe/fitting (20%), and construction (15%) industries.^38^ Magnesium (0.03–0.08 weight percent) is carefully added to molten cast iron during the manufacturing process, changing the shape of graphite from flakes to spheroids and significantly enhancing mechanical qualities.^39,40^

Another attractive and sustainable way to obtain commercial MgO is through MgO-rich solid waste, which is a by-product of ductile cast iron (DCI) production. This waste is dumped in landfills even though it contains a considerable amount of MgO.^38,39,41^ Approximately 500–800 tons of MgO-rich dust are produced annually by a typical ductile iron foundry. This dust is currently managed through landfilling (which costs $50–150 per ton) or stockpiling, which results in resource loss and environmental liability.^34,35^

Literature noticeably lacks systematic research on the valorization of DCI waste for dye removal, despite the significant production of DCI solid waste and the proven effectiveness of MgO-based adsorbents.^26–32^ Steel slag, fly ash, and red mud are the main subjects of current research on metallurgical waste adsorbents; these materials usually need surface modification, thermal treatment, or acid/base activation to function satisfactorily.^25,42,43^ The unique features of DCI waste include highly pure MgO (usually >80%), the intrinsic nanostructure of vapor-condensate, the absence of organic pollutants, and low purchase price, all of which point to extraordinary potential that has not yet been exploited. Moreover, the majority of the previous studies on MgO-based dye removal have focused on laboratory-synthesized materials from chemical precursors or commercially produced MgO,^27–29^ thus providing very little understanding of industrial waste applications. The areas where knowledge is severely lacking are: (1) the relationship between the waste generation conditions and the properties of the adsorbent; (2) the optimization of process parameters specifically for waste-derived adsorbents; (3) performance evaluation in comparison to commercial substitutes;^44,45^ (4) mechanistic understanding of dye waste interactions; and (5) techno-economic feasibility for industrial implementation.

To the best of the authors' knowledge, systematic investigation of DCI foundry dust, which is a high-purity, vapor-condensed MgO by-product to be used as an unmodified adsorbent for cationic dye removal, has not been previously reported. Although MgO sorbent research based on laboratory-prepared or commercially available MgO is abundant, very little work has been done on the use of metallurgical solid waste streams as adsorbents without activation of any kind. This study attempts to fill this knowledge gap through a conceptual demonstration. The present work contributes a proof-of-concept demonstration under realistic process conditions, encompassing: (i) comprehensive physicochemical characterization of the waste material; (ii) Box–Behnken design optimization of adsorption parameters; (iii) equilibrium isotherm, kinetic, and thermodynamic analysis; (iv) post-adsorption mechanistic elucidation *via* XRD, FTIR, and SEM-EDX; (v) 15-cycle regeneration performance assessment; and (vi) preliminary techno-economic feasibility analysis.

## 2 Materials and methods

### 2.1 Materials and reagents

#### 2.1.1 Chemicals and reagents

All chemicals involved in this research were of analytical reagent grade (≥99% purity) and were directly used without any further purification. Crystal Violet (Basic Violet 3) was manufactured by Sigma-Aldrich (St. Louis, MO, USA). Double-distilled conductivity <2 µS cm^−1^ was utilized for the preparation and dilution of all solutions.

#### 2.1.2 Adsorbent material

Solid wastes were sampled from the baghouse filter system in an Egyptian ductile iron foundry (Egyptian Foundry Ltd, Cairo, Egypt). Three shifts' samples were mixed and homogenized through the cone-and-quartering procedure (ASTM D7928-17).^46^ The main benefit of this material is that it was utilized straight off without requiring grinding, sieving (particle size already <200 µm due to vapor condensation), washing, or thermal activation, which resulted in a huge economic saving.

### 2.2 Adsorbent characterization techniques

XRF is used to figure out what chemicals were in the samples. Also, dried samples (2 grams each) are heated to 950 °C for 2 hours to loss on Ignition when burned (LOI).^47^ PANalytical X'Pert Pro MPD powder diffractometer for X-ray Diffraction Analysis (XRD) was used to identify all phases present in the sample.^48^ The particle size distribution and the particle size were determined using Malvern Mastersizer 3000 with Hydro EV wet dispersion unit based on ASTM E 11/2009 and ASTM D 422/2007 (ref. 49) standards. Micromeritics ASAP 2020 surface area and porosity analyzer was used to determine the total pore volume, micropore volume, mesopore volume, pore size distribution, and average pore diameter.^50^ Scanning electron microscopy and energy-dispersive X-ray spectroscopy were used to show the morphology and make the elemental analysis.^51^ Fourier Transform Infrared Spectroscopy (FTIR) was used to determine the functional group present in the samples and the changes that happened to the sample before and after adsorption.^48,52^ Thermogravimetric Analysis (TGA-DTG) to determine the effect of the temperature change and the weight loss happened to the absorbent.^53^ Zeta potential and point of zero charge (pHpr^*c*^) were determined over pH 3–11 using a Malvern Zetasizer Nano ZS.^54^

### 2.3 Batch adsorption experiments

A stock solution of the dye was made by dissolving 1.00 g of crystal violet (CV) in 1000 mL of double-distilled water (1000 mg L^−1^). The stock solution was kept in amber glass bottles, which were wrapped with aluminum foil to prevent decay due to light, and was stored in a refrigerator at 4 °C. Working solutions at a concentration of 5–100 mg L^−1^ were prepared fresh daily from the stock solution. A calibration curve was made in concentration range of 0.5–50 mg L^−1^ and showed very good linearity with correlation coefficients (*R*^2^) higher than 0.9995. Crystal violet has an absorption peak (*λ*_max_) at 590 nm in water at pH 7.0 ± 2.

The batch adsorption tests were carried out in 250 mL Erlenmeyer flasks with 100 mL of CV solution. A temperature-controlled orbital shaker set at 25 ± 1 °C with a preset agitation rate was used for the experiments. The pH was kept at its natural range of 7.0 ± 2. Samples were removed at different intervals, centrifuged for five minutes at 10 000 rpm, and the supernatant was subjected to spectrophotometric analysis (Shimadzu UV-1800) at *λ*_max_ = 590 nm using newly created calibration curves (*R*^2^ > 0.999). Eqn (1) and (2) were used to calculate the removal efficiency and adsorption capacity, respectively.^55^1
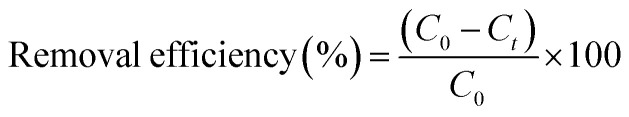
2
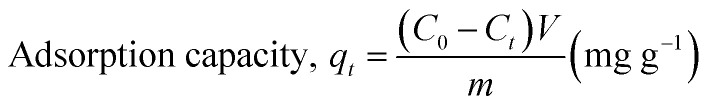
where *C*_0_ = initial concentration (mg L^−1^), *C*_*t*_ = concentration at time *t* (mg L^−1^), *V* = solution volume (L), *m* = adsorbent mass (g).

### 2.4 Experimental design and optimization

Selection of Box–Behnken design over central composite design was done for the following four reasons: (i) the Box–Behnken design requires only 29 trials for four independent variables against 30 trials needed in a face-centered central composite design, giving a slightly higher efficiency in trials; (ii) Box–Behnken design does not generate corner combinations of factors where the independent variables assume extreme values together, which might prove unfeasible or even unsafe in experiments involving absorption studies; (iii) Box–Behnken design is effective in generating unbiased estimates of quadratic coefficients; and (iv) all the trials lie within the safe operating zone of the independent variable space. A response surface methodology based on a Box–Behnken design was carried out using Design-Expert software (Version 13, Stat-Ease Inc., USA).^56^ Box–Behnken design with four parameters at three levels (−1, 0, +1) was employed using *A*: initial concentration (20, 40, 60 mg L^−1^), *B*: adsorbent dose (2, 6, 10 g L^−1^), *C*: stirring rate (150, 250, 350 rpm), and *D*: contact time (0.5, 2.5, 4.5 hours). There were five center point replicates and twenty-four factorial points in the 29 experimental runs that were made up for design generation, ANOVA, model construction, and optimization. The initial dye concentration (20–60 mg L^−1^) is used simulate low-to-moderate pollution loads and represents typical levels seen in textile and dye-containing industrial effluents following primary treatment. Adsorbent dose (2–10 g L^−1^) was selected based on economic viability. Stirring speed (150–350 rpm) was selected to ensure a sufficient level of external mass transfer and a uniform suspension of the adsorbent. Contact time range from 0.5 to 4.5 h, was chosen to ensure complete cover equilibrium stage.

### 2.5 Adsorption isotherm studies

The adsorption isotherms were conducted under the optimized parameters obtained from Box–Behnken design by varying the initial dye concentration (5–100 mg L^−1^). Both linear and non-linear regression were used to fit the data into four isotherm models: Langmuir, Freundlich, Temkin, and Dubinin–Radushkevich. To clarify the adsorption mechanism and surface properties of the adsorbent, dye adsorption behavior was examined by fitting the experimental equilibrium data to many adsorption isotherm models. Monolayer adsorption on a homogeneous surface was described by the Langmuir isotherm, which was also used to estimate important parameters, including the separation factor to evaluate adsorption favorability, the maximum adsorption capacity, and the adsorption energy. Multilayer adsorption on heterogeneous surfaces was taken into consideration using the Freundlich model, whose constants represented surface heterogeneity and adsorption capacity. Adsorbent–adsorbate interactions were assessed using the Temkin isotherm, which assumes a linear decrease in adsorption heat with increasing surface coverage. Furthermore, by estimating the mean free adsorption energy, the Dubinin–Radushkevich isotherm was used to differentiate between chemical and physical adsorption, offering a greater understanding of the characteristics of dye adsorption onto the adsorbent. These isotherm models' theoretical foundation and formulation have been documented in earlier research.^57–60^

### 2.6 Adsorption kinetics studies

The adsorption kinetics were conducted under the optimized parameters obtained from the Box–Behnken design with different adsorption times up to 360 minutes. The obtained kinetics data were fitted to different kinetic models; pseudo-first-order, pseudo-second-order, Elovich, Weber–Morris (Intraparticle Diffusion), and Boyd models. To determine the rate-controlling stages and adsorption process, dye adsorption kinetics were assessed by fitting the experimental data to five popular kinetic models. The pseudo-second-order model was used to indicate chemisorption that involves electron sharing or transfer between dye molecules and the adsorbent surface, while adsorption limited by the availability of vacant surface sites was represented by the pseudo-first-order model. Considering chemisorption on heterogeneous surfaces, the Elovich model was used, which assumed that the activation energy decreases as surface coverage increases. The Weber–Morris intraparticle diffusion model was used to decide whether dye diffusion inside the adsorbent pores is the rate-limiting step or if adsorption proceeds through several mass-transfer stages. Additionally, the use of the Boyd model to distinguish between intraparticle diffusion and film diffusion mechanisms resulted in a more profound understanding of the predominant mass-transfer processes governing dye adsorption. The theoretical basis and practical applications of these kinetic models have been comprehensively covered in previous studies.^57–60^

### 2.7 Thermodynamic studies

The effect of temperature on the adsorption was investigated under the optimized parameters obtained from the Box–Behnken design at different temperatures; 288, 298, 308, and 318 K. The distribution coefficient, Gibbs free energy, enthalpy, entropy, and Arrhenius activation energy were calculated. The feasibility, heat effect, and randomness degree of the adsorption process were evaluated through the thermodynamic parameters that is Gibbs free energy (Δ*G*°), enthalpy change (Δ*H*°), and entropy change (Δ*S*°). The unitless distribution coefficient *K*_d_ was obtained using the formula below:*K*_d_ = (*q*_e_/*C*_e_) × *ρ*_solution_ × 1000where *q*_e_ [mg g^−1^] is the amount adsorbed at equilibrium, *C*_e_ [mg L^−1^] is the concentration at equilibrium in the liquid phase, and *ρ*_solution_ is the density of the solution (1.0 g mL^−1^). By multiplying the value of *K*_d_ by *ρ*_solution_ and 1000 mL L^−1^, we obtained a dimensionless distribution coefficient that can be used for van't Hoff analysis as proposed by Milonjić (2007)^61^ and Anastopoulos and Kyzas (2016).^62^ The standard thermodynamic quantities (Δ*G*°, Δ*H*°, Δ*S*°) were obtained by plotting ln *K*_d_*vs.* 1/*T* on a van't Hoff plot. Then, the standard thermodynamic relations were applied to get Δ*G*°. Δ*H*° and Δ*S*° were later obtained by using the van't Hoff method with the help of the linear plot's slope and interception. If the adsorption process is spontaneous, endothermic (positive Δ*H*°) or exothermic (negative Δ*H*°), and if disorder at the solid–liquid interface grows during adsorption can all be determined by analyzing these characteristics. Positive Δ*S*° values show more randomness at the adsorption interface, whereas negative Δ*G*° values show a spontaneous process, with more negative values indicating stronger spontaneity. The thermodynamic models and computation techniques used in this investigation have been extensively documented in earlier research^63,64^

### 2.8 pH effect studies

The effect of solution pH on CV elimination was investigated over the pH range of 3 to 11. The initial pH was changed using either 0.1 M HCl or 0.1 M NaOH. The tests employed *C*_0_ = 40 mg L^−1^, dosage = 6.2 g L^−1^, stirring = 250 rpm, temperature = 25 °C, and contact duration = 4 h. CV speciation at different pH levels was calculated using the Henderson–Hasselbalch equation while accounting for p*K*a values. Crystal violet is a cationic dye that always keeps its positive charge over a wide pH range. At acidic pH, removal efficiency is lowered because of competition between CV cations and H^+^ ions for the limited adsorption sites. Increasing the pH results in a stronger electrostatic attraction and the deprotonation of the adsorbent surface, therefore, raising the incorporation of CV. The dye speciation at different pH values was figured out by using the Henderson–Hasselbalch equation and the CV p*K*a values, and these results were interpreted in terms of the pH-dependent adsorption mechanism.^65,66^

### 2.9 Regeneration and reusability studies

#### 2.9.1 Regeneration method selection

In order to improve the adsorbent performance, several regeneration methods were explored. Acid regeneration took place with the aid of 0.1 M HCl at a temperature of 25 °C and stirred at 300 rpm, a solid-to-liquid ratio of 1 : 20 (w/v), and a time of contact of 30 minutes. Under the same circumstances, base regeneration employed 0.1 M NaOH. 99.9% ethanol was used for organic solvent regeneration under the same circumstances. In thermal regeneration, the adsorbent was heated at a rate of 10 °C per minute for two hours at 300 °C in an air-filled muffle furnace. Additionally, a four-step mixed acid-thermal regeneration approach was tested: four steps made up the chosen combined acid-thermal regeneration protocol, which was applied consistently over all 15 cycles: (a) acid wash (0.1 M HCl, 30 min, 25 °C, solid-to-liquid ratio 1 : 20 (w/v), 300 rpm); (b) washing (deionized water wash until pH 6–7); (c) drying (105 °C overnight); and (d) thermal treatment 300 °C for two hours in the air. The desorption efficiency was calculated using eqn (3).3
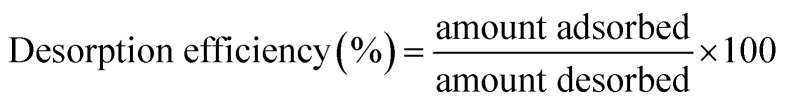


#### 2.9.2 Long-term reusability (15 cycles)

The adsorbent was tested for 15 consecutive cycles under the following adsorption conditions: pH 8.0, temperature 25 °C, stirring at 250 rpm, CV concentration of 40 mg L^−1^, adsorbent dose of 6.2 g L^−1^ (reusing the same adsorbent), and contact period of 4 hours. Following the foregoing procedure, the combined acid-thermal method, which was found to be the best-performing approach during screening, was used for regeneration following each cycle. Capacity retention was used to calculate performance metrics using eqn (4).4
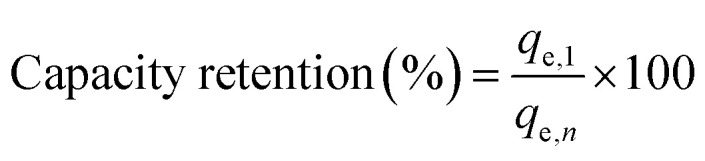
where *q*_e,*n*_is the equilibrium capacity at cycle *n*, and *q*_e,1_is the equilibrium capacity of the fresh adsorbent.

### 2.10 Heavy metal leaching assessment

By interacting DCI waste (5 g L^−1^) with deionized water through 300 rpm mixing at different pH values, temperatures, and contact time, potential metal leaching under worst-case scenarios, as shown in Table 1 was assessed and measured by ICP-OES (PerkinElmer Optima 8000) after filtered samples were acidified with 2% HNO_3_. The results were compared to USEPA,^67^ WHO,^68^ and Egyptian Environmental Law no. 4/1994 (ref. 69) drinking water guidelines.

**Table 1:** Test conditions (worst-case scenarios)

pH	Temperature (°C)	Contact time (h)	Rationale
3.0	45	24	Acidic industrial effluent, elevated temperature, and extended contact
5.0	45	24	Slightly acidic, worst-case operational
7.0	45	24	Neutral pH, elevated temperature
8.0	25	4	Normal operational conditions (baseline)
9.0	45	24	Alkaline, elevated temperature
11.0	45	24	Strongly alkaline, worst-case

### 2.11 Economic analysis

For a medium-sized wastewater treatment facility with a design capacity of 1000 m^3^ per day that operates 365 days a year, a thorough techno-economic analysis was carried out. To ascertain total economic viability under conditions of continuous industrial operation, the study considered both capital and operating costs. All of the initial investments needed to put the adsorption system into place were covered by capital expenditures. The acquisition of process equipment, such as adsorption tanks, pumps, pipes, and control systems, as well as related civil works, such as foundations, structural elements, and electrical connections, was all included in this. To represent reasonable implementation costs, installation and commissioning tasks were also included. All ongoing expenses incurred during plant operation were included in operational expenditures. Adsorbent consumption, while taking up to 15 cycles of reuse into account, energy consumption for mixing, pumping, and heat activation, chemical reagents needed for adsorbent regeneration, labor needs, regular maintenance, and waste handling and disposal expenses were all considered. The unit treatment cost, which was stated in US dollars per cubic meter, was determined by dividing the annual total cost by the volume of treated wastewater. The ratio of capital expenditure to the yearly cost reductions attained as compared to traditional treatment technologies was used to evaluate the payback period. The effectiveness and financial viability of the DCI waste-derived adsorbent were compared to commonly used commercial materials through a comparative cost analysis. Activated carbon derived from coal and coconut shells, activated alumina, commercial calcined magnesium oxide, bentonite modified with lanthanum, ion exchange resins, and granular ferric hydroxide were all compared.

## 3 Results and discussion

### 3.1 Comprehensive characterization of DCI solid waste

#### 3.1.1 Chemical composition (XRF analysis)

The elemental composition of DCI solid waste was determined by X-ray fluorescence spectroscopy (Table 2), which showed a very high MgO content (88.0 wt%) and very low quantities of other metal oxides. The high melting temperature of ductile cast iron (1450–1500 °C), which results in significant magnesium evaporation and oxidation, is the cause of its extraordinary purity.^70^ Filtration devices collect MgO fumes produced by the breakdown of magnesium-cored wire structure in the foundry atmosphere.

**Table 2:** Chemical composition of DCI solid waste determined by XRF

Oxide	Percentage (%)
**MgO**	**88**
Fe_2_O_3_	2.28
ZnO	4.2
Na_2_O	0.4
SiO_2_	0.2
CaO	0.24
MnO	0.04
TiO_2_	0.02
K_2_O	0.01
P_2_O_2_	0.01
**L.O.I**	**4.54**

Zinc (4.2 wt% ZnO) and iron oxides (2.28 wt% Fe_2_O_3_) with trace amounts of aluminum oxide (0.86 wt% Al_2_O_3_) are produced due to the oxidizing environment and high temperatures during manufacture. The presence of volatile components, mainly carbonates and hydroxides produced by ambient CO_2_ and moisture interaction with basic MgO surfaces during storage, is shown by the loss on ignition (4.54%).

The amphoteric nature of MgO allows for effective operation over a wide pH range (5–10) by generating positively charged (Mg–OH_2_^+^) or negatively charged (Mg–O^−^) surface species depending on solution conditions promoting strong electrostatic attraction toward cationic dyes like crystal violet (CV^+^), and its ionic character and defect-rich structure provide many coordinatively unsaturated sites that improve surface reactivity and molecular adsorption. Minor components also enhance performance: ZnO provides amphoteric adsorption sites that are effective at near-neutral pH, and Fe_2_O_3_ contributes additional active sites through surface complexation. The low loss on ignition indicates good thermal stability, which is beneficial for regeneration processes.^71^

#### 3.1.2 Mineralogical analysis (XRD)

**Fig. 1:**
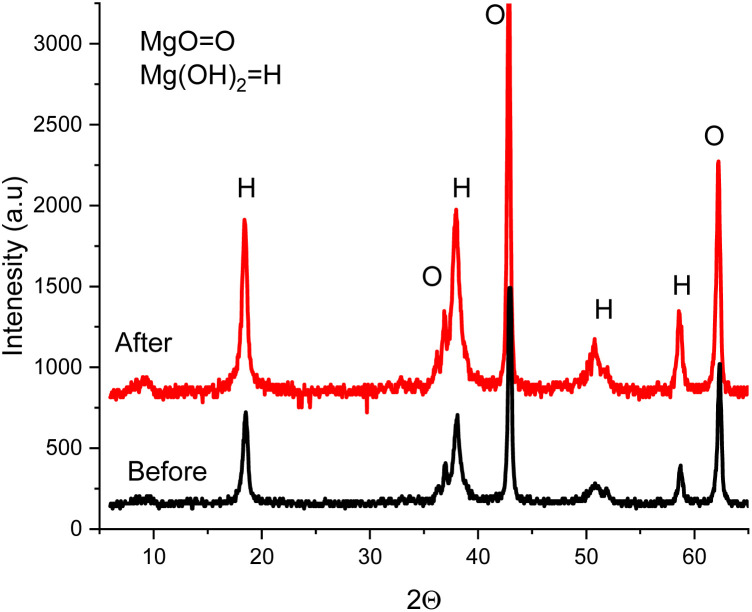
XRD patterns for the DCI sample prior to and after the adsorption process.

X-ray diffraction analysis was used to identify crystalline phases present in DCI solid waste using the ICDD database^72^ (Fig. 1). The XRD pattern of fresh DCI shows sharp, distinct diffraction peaks, indicative of a highly crystalline material with minimal amorphous content. The primary phase of the material is periclase (MgO, PDF #45-0946). The main crystalline phase has characteristic diffraction peaks at 2*θ* = 36.9°, 42.9°, 62.3°, 74.7°, and 78.6°, which correspond to the (111), (200), (220), (311), and (222) Miller planes of the cubic periclase structure. Based on quantitative analysis of Rietveld refinement, zincite (ZnO): 5.8 ± 0.8 wt%, hematite (Fe_2_O_3_): 2.2 ± 0.4 wt%, periclase (MgO): 91.3 ± 2.1 wt%, and amorphous content: 0.7 ± 0.3 wt%. These results have a strong correlation with XRF elemental analysis. Zinc impurities in cored wire or molten iron are the source of one of the minor phases, Zincite (ZnO, PDF #36-1451), which displays weak peaks at 2*θ* = 31.8°, 34.4°, and 36.3°, corresponding to a hexagonal wurtzite structure. The second phase is hematite (Fe_2_O_3_, PDF #33-0664), which has very weak reflections at 2*θ* = 33.2° and 35.6° that show the interaction of steel sheathing residues with trace iron oxide from the foundry atmosphere. The sole existence of periclase (cubic MgO) in the absence of magnesium hydroxide [Mg(OH)_2_, brucite] or magnesium carbonate [MgCO_3_, magnesite] phases indicates that waste was appropriately stored in dry conditions to avoid hydration and carbonation. This “as-produced” periclase offers a major financial benefit since it doesn't need to be chemically or thermally pretreated before adsorption applications.

Post-adsorption XRD analysis (Fig. 1) showed crucial structural transformation. Magnesium hydroxide [Mg(OH)_2_, Brucite, PDF #44-1482] formed a new phase with additional diffraction peaks at 2*θ* = 18.6°, 38.0°, 50.9°, and 58.6°, which corresponded to the (001), (101), (102), and (110) planes of the hexagonal brucite structure. From the quantitative Rietveld refinement, MgO content was seen to reduce from 91.3 ± 2.1 wt% to 69.5 ± 2.3 wt%, with 22.1 ± 1.8 wt% of Mg(OH)_2_ being formed due to aqueous contact. Notably, a blank test involving contact between DCI waste and deionized water under the same conditions (pH 8.0, 25 °C, 4 hours, and 6.2 g L^−1^) but without CV resulted in 22.3 ± 1.9 wt% of Mg(OH)_2_. This observation means that the conversion of MgO to Mg(OH)_2_ through hydration (MgO + H_2_O − > Mg(OH)_2_) is not related to dye molecules, implying that Mg(OH)_2_ cannot be used as direct evidence to confirm CV adsorption. However, the higher Mg(OH)_2_ content will mean more hydroxyl groups on its surface available for CV hydrogen bonding, while a slight pH rise during adsorption suggests that the reaction involves OH^−^ formation. The fact that Mg(OH)_2_ is reversible by 300 °C heating makes adsorbent regeneration possible. It is worth noting that there were no CV diffracted peaks in the XRD pattern of the adsorbed dye, suggesting it is either amorphous or under the detection limit of about 3 wt%.

One of the critical facts is that Mg(OH)_2_ causes a pH level increase during the adsorption process, but this increase only goes as high as ∼8.0–8.5, which is way under the CV p*K*a of 9.4. At this pH, CV speciation calculations (Section 3.6.3) have shown that 96.2% of the dye molecules are still in the colored cationic form CV^+^. Thus, the color disappearance that was seen is the result of a genuine drop in the amount of substance in solution through adsorption onto the solid phase and is not the result of dye decolorization through carbinol (CVOH) formation due to increased alkalinity. This has been confirmed by the following evidences: (1) post-adsorption EDX analysis detecting nitrogen (2.2 at%) and chlorine (1.2 at%) on the isolated solid adsorbent; (2) FTIR revealing typical CV aromatic peaks on the solid phase; (3) BET pore volume decreased by 0.034 cm^3^ g^−1^, which signifies that the pores are physically occupied; and (4) 95.4% of the dye being recovered from the solid adsorbent by acid-thermal regeneration.

#### 3.1.3 Surface area and pore structure (BET analysis)

**Fig. 2:**
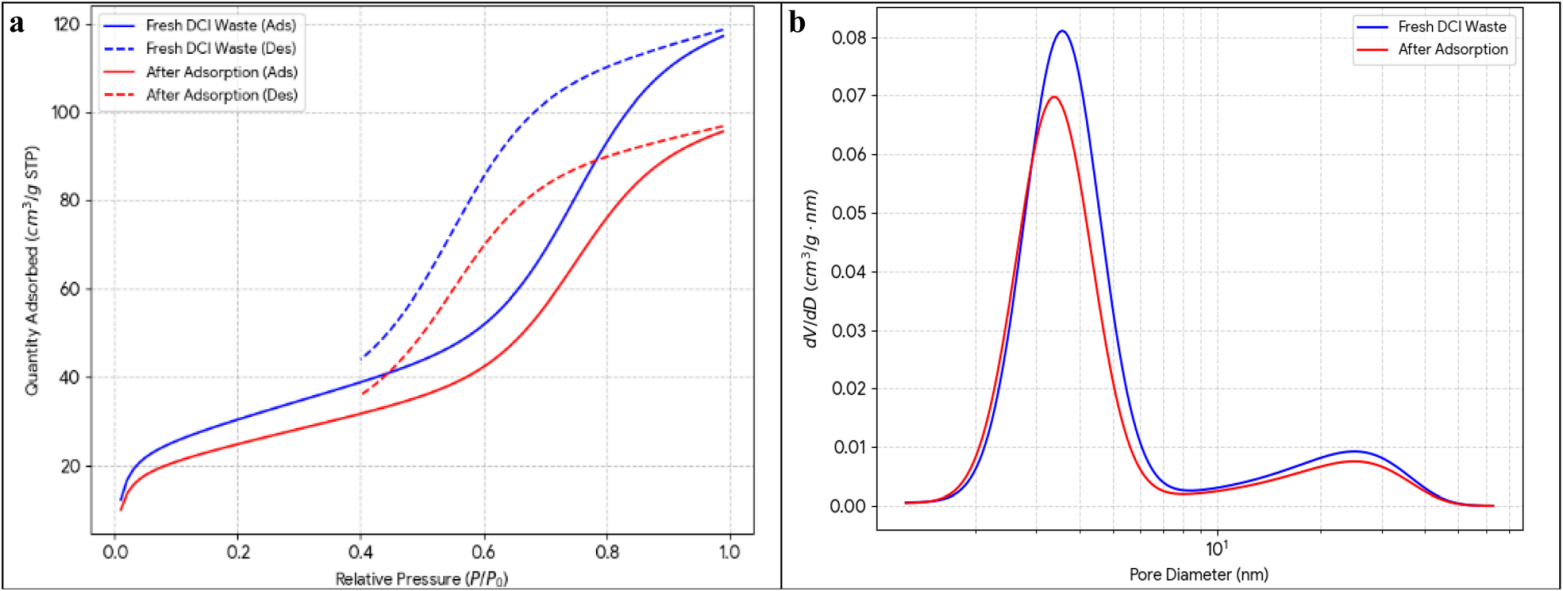
The BET resulted graphs. (a) Nitrogen adsorption–desorption isotherms. (b) BJH pore size distribution.

Through nitrogen adsorption–desorption isotherms at 77 K (Fig. 2 and Table 3), key textural properties influencing adsorption efficiency were identified.

**Table 3:** BET surface area and porosity characteristics

Parameter	Fresh DCI waste	After adsorption	Change
BET surface area (m^2^ g^−1^)	247 ± 5	201 ± 4	−18.6%
Total pore volume (cm^3^ g^−1^)	0.185	0.151	18.4%
Micropore volume (cm^3^ g^−1^)	0.038	0.029	−23.7%
Mesopore volume (cm^3^ g^−1^)	0.147	0.122	−17.0%
Average pore diameter (nm)	3.8	3.6	−5.3%
External surface area (m^2^ g^−1^)	198	163	−17.7%

The N_2_ adsorption–desorption isotherm (Fig. 2a and b) followed type IV with an H3 hysteresis loop pattern, indicative of mesoporous structure with slit-like pores; see SI, S1 for detailed explanation of IUPAC classification.^73^

One of the main factors determining the adsorption of crystal violet by an adsorbent is the adsorbent's textural properties, chiefly, the very high specific surface area of 247 m^2^ g^−1^ that offers an incredibly large number of active sites for the dye to bind, thus completely outclassing most adsorbents derived from agricultural waste (generally 50 150 m^2^ g^−1^)^74,75^ and nearness to the performance of commercial activated carbons (400 1200 m^2^ g^−1^). It is especially interesting to note that this was achieved without chemical activation, and only mild thermal treatment was used. The size of the crystal violet molecules fits perfectly with the predominance of *meso*-porosity (79.5%), the pore diameters being mainly in the 3–12 nm range. This facilitates fast diffusion and thus, the typical problems of steric hindrance and diffusion resistance, which are caused by micropores, are avoided, hence the explanation of the rapid adsorption kinetics and getting to the equilibrium within 4 hours.

Following crystal violet uptake, post-adsorption BET analysis of the adsorbent shows a dramatic reduction of its features of textural nature, in particular, the specific surface area has decreased from 247 to 201 m^2^ g^−1^ (18.6%), which is confirmation of pores being occupied by adsorbed molecules of the dye. Like this, the total pore volume of the adsorbent has decreased from 0.185 to 0.151 cm^3^ g^−1^ (18.4%), which is approximately 0.034 cm^3^ g^−1^ of pore volume occupied by dye.^76^ However, while the larger mesopores maintain the accessible surface area, the micropore volume shows an even greater loss (23.7%) compared to mesopores (17.0%), implying that smaller pores, which have higher adsorption potentials, better fit preferential filling or blockage, act as adsorption sites that more complete occupation is achieved.

#### 3.1.4 Particle size distribution

**Fig. 3:**
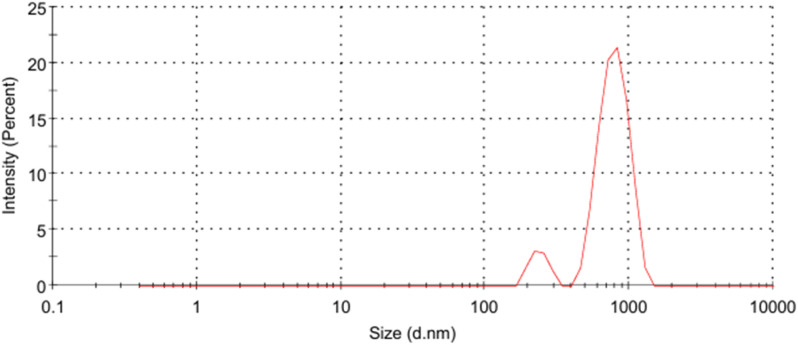
Particle size distribution by laser diffraction.

Reduction of particle size distribution to submicron obtained with narrow polydispersity was confirmed by laser diffraction analysis (Fig. 3 and Table 4).

**Table 4:** Particle size distribution parameters

Parameter	Value	Significance
*D*_10_ (µm)	0.052	10% volume below 52 nm
*D*_50_ (µm)	0.098	Median diameter 98 nm
*D*_90_ (µm)	0.187	90% volume below 187 nm
Mean diameter (µm)	0.106	Volume-weighted average

Almost all particles (85%) are found in the range 40–150 nm, while only a few fine and coarse particles are present. The particle size distribution is monomodal. The material has a very high surface area due to its median particle size of 98 nm, which puts it in the nanoparticle range. This is supported by the close match between the measured BET value of 247 m^2^ g^−1^ and the predicted geometric surface area of roughly 252 m^2^ g^−1^. The high particle number density, estimated at 8.2 × 10^12^ particles per gram, boosts contact frequency with adsorbate molecules. Particles below 200 nm show good suspension stability, but some aggregation occurs, reflected by moderate polydispersity with a span of 1.38 and *D*_90_ of 187 nm, consistent with SEM observations. Solid–liquid separation requires centrifugation rather than gravity settling. The nanoscale size originates from vapor-phase nucleation and growth of MgO during magnesium oxidation at 1450–1500 °C, followed by rapid cooling that preserves the submicron particle size.

#### 3.1.5 Surface morphology and elemental distribution (SEM-EDX)

**Fig. 4:**
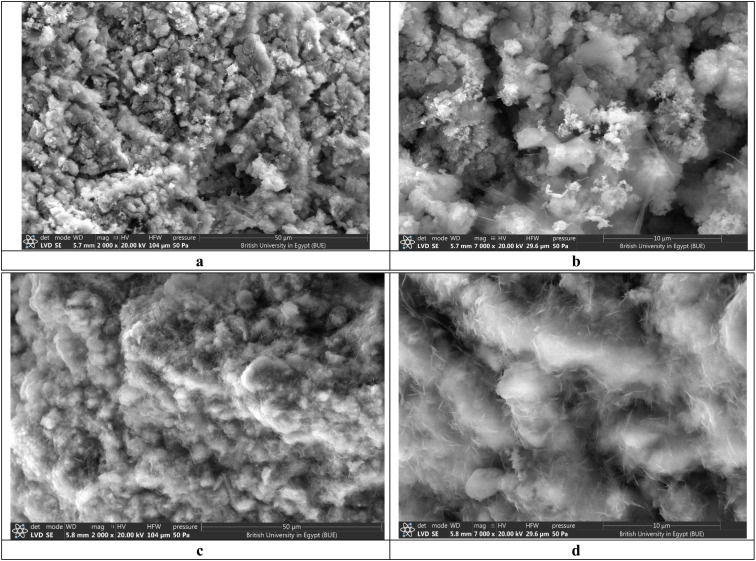
SEM morphological analysis of DCI adsorbent: (a and b) before adsorption; (c and d) after adsorption.

Scanning electron microscopy (Fig. 4a and b) images illustrate the DCI waste powder as a combination of irregularly shaped secondary agglomerates of metal oxide nanoparticles that are held tightly together by strong interparticle van der Waals and electrostatic forces. When further zoomed in, the agglomerates show rough, heterogeneous surfaces with an abundance of cracks and crevices, thus forming a highly porous structure that allows the fluid to easily penetrate internal voids. High-resolution SEM images display a highly permeable microstructure with an interconnected mesoporous network. The agglomerates consist of primary nano-crystallites with diameters of 30–80 nm (which is in line with XRD crystallite size analysis) that self-assemble into larger secondary particles with a median size of 98 nm as confirmed by laser diffraction particle size distribution (*D*_50_ = 0.098 µm, spanning 52–187 nm for *D*_10_–*D*_90_ range). Energy-dispersive X-ray spectroscopy (EDX) reveals that magnesium (39.5 at%) and oxygen (52.3 at%) are the major elements on the surface, along with a small amount of zinc (5.2 at%) and iron (2.4 at%), which agrees with an MgO-rich matrix. The extraordinarily irregular pore surface morphology revealed at high magnification is the reason for the extensive specific surface area of 247 m^2^ g^−1^, which was measured by BET analysis. BJH pore size distribution analysis shows a multimodal mesoporous structure where the primary pore population is at 3.5–4.2 nm (making up ∼60% of total pore volume) with one secondary mesopore population being extended to 30–40 nm from interparticle voids. Hence, this hierarchical porous architecture offers a plethora of readily accessible, high-energy adsorption sites that make a significant contribution to the material's excellent capacity for crystal violet removal.

Post-adsorption SEM images (Fig. 4c and d) revealed significant morphological changes, which visually confirmed crystal violet adsorption on DCI. The originally rough surfaces appear to be partially covered with new organic deposits, which were identified as adsorbed dye molecules together with some water. At higher magnifications, it may be seen that the mesopores are partly filled and the pore openings are reduced, which is in line with the experimental data that reported a decrease in the BET surface area of 18.6% from 247 to 201 m^2^ g^−1^. EDX analysis after the adsorption process reveals very significant changes in surface composition: nitrogen (2.2 at%) and chlorine (1.2 at%) peaks, which are the main features of crystal violet molecules and their chloride counter-ion, respectively, appear; and carbon content increases five times (from 0% to 4.1 at%) due to the presence of the organic dye structure. The signals for magnesium and oxygen remain, which means that the MgO-based adsorbent matrix has kept its structure. Textural analysis suggested that the smaller pores were selectively occupied because the micropore volume showed a greater reduction (23.7%) than the mesopore volume (17.0%), which means that the high-energy sites in the smaller pores are the first to be filled during the adsorption process. Although its surface has been modified, the material still has a hierarchical porous structure, and the interconnected pore networks are still accessible, which means that it can be regenerated and reused several times.^77–81^

**Fig. 5:**
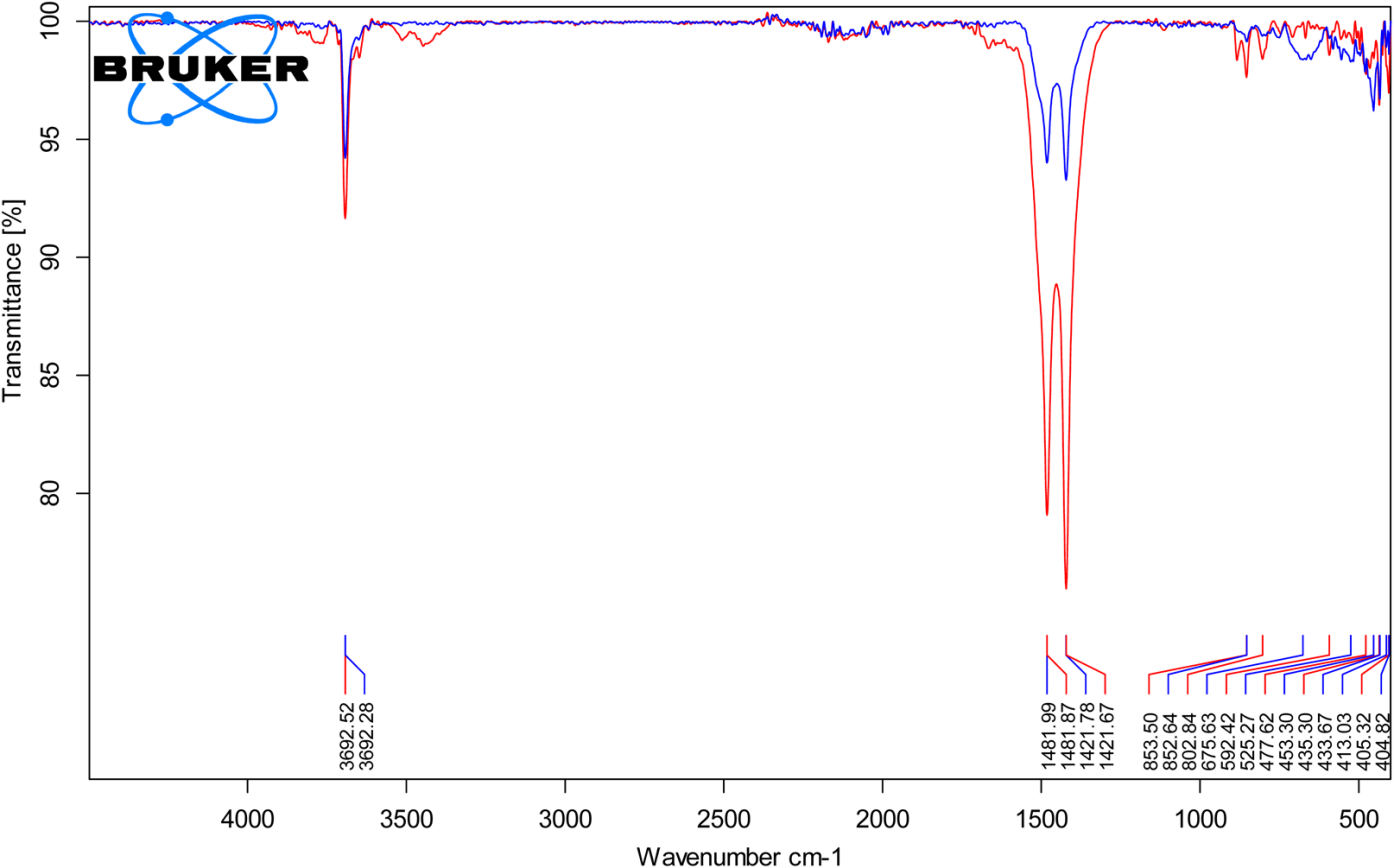
FTIR spectrum before adsorption (blue), and after adsorption (red).

EDX analysis to adsorption (Fig. 5a) verifies that the surface of the material mainly comprises oxygen and magnesium, with the major peaks of O Kα and Mg K_α_/K_β_ indicating an MgO-rich matrix, while moderate zinc peaks and weak iron and aluminum peaks point to minor surface-enriched phases, and carbon is only present at very low levels due to atmospheric contamination with no nitrogen detected. Quantitative surface determination of the elements reveals that oxygen and magnesium are the major ones, followed by zinc, iron, and trace amounts of aluminum, with good agreement between point readings.

EDX investigation after adsorption (Fig. 5b) presents conspicuous changes in elements that undoubtedly correspond to crystal violet absorption along with preservation of the MgO-based matrix beneath. Arising from the CV molecule and its chloride counter-ion, new nitrogen and chlorine peaks are noticeable while carbon intensity rises more than five times due to the organic dye, still, Mg, O, Zn, Fe, and Al peaks are there, pointing to the structural integrity of the adsorbent. It is found that 2.2 at% nitrogen and 1.2 at% chlorine correspond to an estimated CV surface loading of about 12.6 wt% within the EDX sampling depth, which is in line with monolayer to few-layer coverage. The rise in oxygen content is attributed to surface hydroxylation and adsorbed water, which is consistent with partial MgO conversion to Mg(OH)_2_ observed by XRD. The apparent decreases in metal contents can be explained by the dilution effect caused by the additional organic layer rather than the metal being lost (Table 5).^77–81^

**Table 5:** EDX results before and after adsorption

EDX before adsorption			EDX after adsorption			
Element	Atomic %	Weight %	Element	Atomic %	Weight %	Change, Wt. %
O	52.3	35.1	O	55.8	38.2	+3.1
Mg	39.5	40.2	Mg	36.2	37.6	−2.6
Zn	5.2	14.3	Zn	4.8	13.4	−0.9
Fe	2.4	5.6	Fe	2.2	5.3	−0.3
Al	0.6	0.7	Al	0.5	0.6	−0.1
C	0.0	0.1	C	4.1	2.1	+2
			N	2.2	1.3	+1.3
			Cl	1.2	1.8	+1.8

#### 3.1.6 Fourier transform infrared spectroscopy (FTIR)

**Fig. 6:**
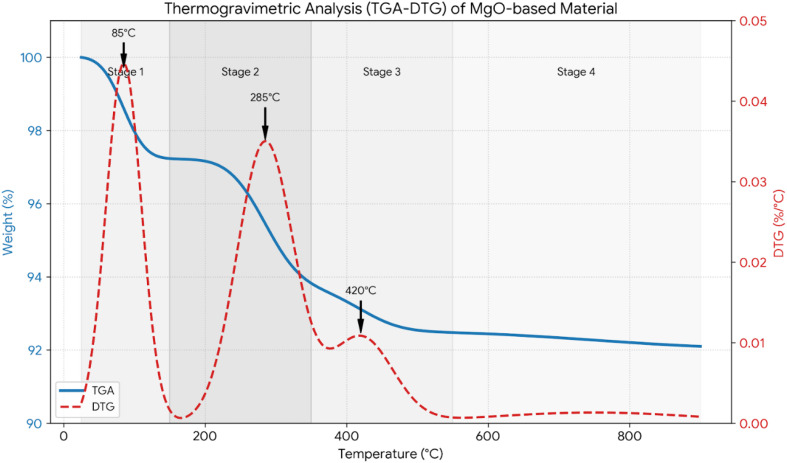
Thermogravimetric analysis (TGA-DTG).

Looking at this FTIR spectrum (Fig. 6) FTIR analysis of DCI waste before and after adsorption (Fig. 5) reveals the following key spectral changes. This represents free O–H stretching vibrations from surface hydroxyl groups (Mg–OH) on the MgO surface. The slight shift and intensity reduction after adsorption suggests possible hydrogen bonding interactions between crystal violet and the surface hydroxyl groups. For Mid-IR region (∼1481–1421 cm^−1^) the pre-adsorption spectrum shows relatively flat baseline with minimal absorption with post-adsorption spectrum shows dramatic appearance of strong, sharp peaks at approximately 1481.99 cm^−1^, 1481.87 cm^−1^, 1421.78 cm^−1^, and 1412.67 cm^−1^. These peaks are characteristic of crystal violet dye structure as at ∼1481 cm^−1^ there is aromatic C=C stretching vibrations from the three benzene rings in crystal violet while at ∼1421–1412 cm^−1^ there are C–H in-plane bending vibrations, aromatic ring stretching, and possible C–N stretching from the central tertiary amine group. The intensity of these peaks provides strong evidence of substantial dye loading on the MgO surface.

A fingerprint region (∼853–405 cm^−1^) with multiple new/enhanced peaks appear after adsorption at ∼853.50 cm^−1^ this could represent aromatic C–H out-of-plane bending, at ∼802.84 cm^−1^ occurs because of aromatic ring breathing modes, at ∼762.83 cm^−1^ because of *para*-substituted benzene rings (crystal violet contains dimethylamino groups in *para* positions), at ∼592.42 cm^−1^ because of C–N stretching vibrations from aromatic amines at ∼527.42 cm^−1^, 517.62 cm^−1^ means possible *N*-phenyl vibrations, at ∼453.30 cm^−1^ may indicate Mg–O vibrations or interactions, and at ∼433.67–404.82 cm^−1^ this region could represent Mg–O lattice vibrations from the MgO adsorbent, or possible formation of surface complexes between dye and MgO. The MgO surface characteristics important for this analysis are the basic surface nature as MgO is a basic oxide (Lewis base), which can interact with the cationic crystal violet dye (a triphenylmethane dye with positive charge), surface hydroxyl groups as MgO readily forms Mg–OH groups in aqueous solutions, and Electrostatic interactions as the positively charged crystal violet cation should strongly adsorb onto the negatively charged or hydroxylated MgO surface.

The FTIR data suggests multiple interaction mechanisms such as electrostatic attraction due to the appearance of intact aromatic peaks suggests the dye structure is preserved, indicating surface adsorption rather than chemical degradation, or hydrogen bonding due the shift in the 3692 cm^−1^ peak suggests H-bonding between surface Mg–OH groups and the dye molecules, or surface complexation due to the changes in the low-frequency region (<500 cm^−1^) may indicate direct coordination between the dye and Mg sites on the surface. The dramatic decrease in transmittance (increase in absorbance) in the post-adsorption spectrum across the 1500–400 cm^−1^ region indicates high surface coverage of crystal violet, strong retention of dye on the MgO surface, and successful wastewater treatment with significant dye removal. The FTIR analysis confirms highly effective adsorption of crystal violet dye onto the ductile cast iron (MgO) adsorbent. The preservation of characteristic aromatic and amine functional groups indicates physical/electrostatic adsorption as the primary mechanism, with possible hydrogen bonding contributions. Strong interactions between the MgO's basic surface properties and surface hydroxyl groups with the cationic dye are the factors which lead the MgO to be an excellent adsorbent for crystal violet removal from wastewater. The appearance and/or increase in the peak intensities at 1481–1421 cm^−1^ is an indication of a high adsorption capacity, thus this material can be very useful for the industrial wastewater treatment processes.^52^

#### 3.1.7 Thermogravimetric analysis (TGA-DTG)

The weight loss of 8.0% from 25 to 900 °C based on the TGA-DTG results show that the DCI waste has excellent thermal stability. The two major thermal events occur at 85 °C (loss of physisorbed water) and 285 °C (dehydroxylation) as shown in Fig. 6. These events confirm the choice of regeneration and thermal activation temperatures (300 and 400 °C). Details of the TGA stages are discussed in SI, Section S2.^82–84^

### 3.2 Response surface methodology optimization

The experiment was divided into two consecutive stages: In Stage 1, the Box–Behnken design of RSM was employed to determine the optimum operating parameters of CV removal (Section 3.2), while in Stage 2, the optimized operating parameters were kept constant, and the mechanism study was performed by measuring the isotherm, kinetics, thermodynamic parameters, and adsorption product characterization (Sections 3.3 to 3.6). By methodically examining four operational parameters that affect CV removal efficiency, Box–Behnken design uncovered intricate relationships that are impossible to find using conventional one-factor-at-a-time methods.

#### 3.2.1 Model development

The simplified quadratic model (eqn (5)) was obtained by first fitting a quadratic model and then backward eliminating non-significant terms (*p* > 0.10):

5 *Y* = 0.89*A* + 18.15*B* + 0.49*C* + 21.35*D* − 2.47*BD* − 0.012*A*^2^ − 1.37*B*^2^ − 0.001*C*^2^ − 1.94*D*^2^

where *Y* = dye removal efficiency (%), *A* = initial concentration (mg L^−1^), *B* = adsorbent dose (g L^−1^), *C* = stirring rate (rpm), *D* = contact time (h).

Excellent model significance was shown by Analysis of Variance (ANOVA) (Table 6).

Reduced quadratic model

**Table:** 

ANOVA analysis			
Source	*F*-value	*p*-value	Significance
Model	66.83	< 0.0001	**Highly significant**
*A*-Initial concentration	0.3583	0.5584	Not significant
*B*-Adsorbent dose	1.01	0.03306	Significant
*C*-Stirring rate	0.0163	0.9002	Not significant
*D*-Time	1.76	0.02044	Significant
*BD*	149.56	< 0.0001	**Highly significant**
*A*2	10.11	0.0062	Significant
*B* ^2^	55.87	< 0.0001	**Highly significant**
*C* ^2^	12.12	0.0033	Significant
*D* ^2^	51.62	< 0.0001	**Highly significant**

**Table:** 

Model adequacy statistics	
*R* ^2^	0.9757
Adjusted *R*^2^	0.9611
Predicted *R*^2^	0.9155

**Fig. 7:**
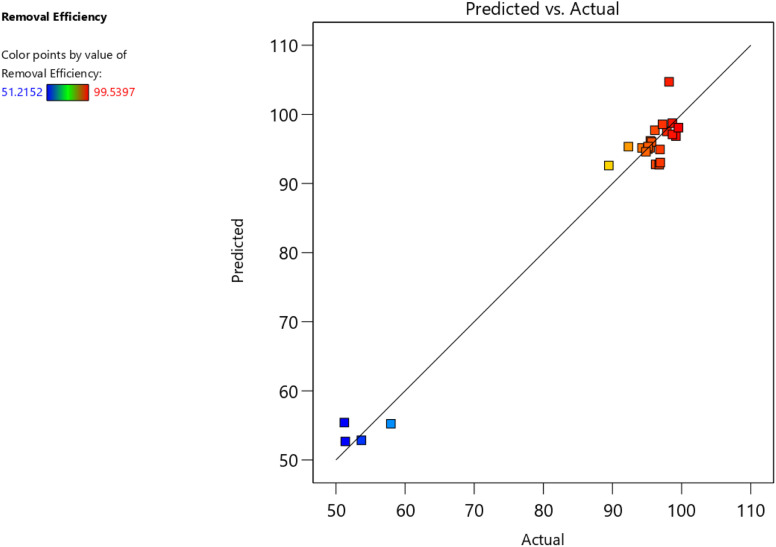
The link between the predicted and experimental dye removal efficiency.

The predicted *vs.* actual values plot (Fig. 7) shows points clustering along 45° line with minimal scatter, confirming model predictive capability.

#### 3.2.2 Individual parameter effects

**Fig. 8:**
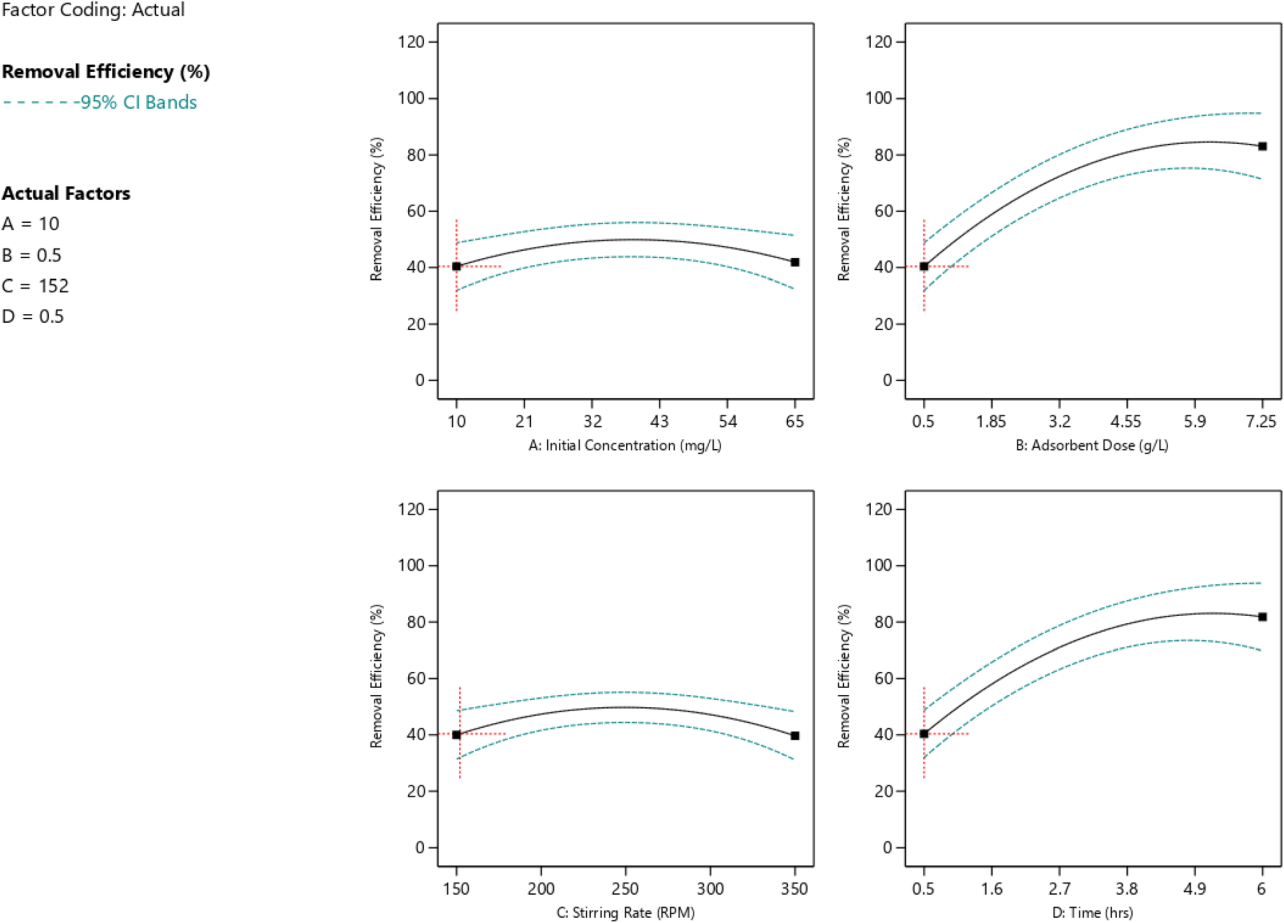
The impact of all process parameters on dye removal %.

Based on Fig. 8, with a *p*-value of 0.0204, contact time exhibits moderate significance. Removal efficiency exhibits a three-phase pattern, increasing from roughly 55% at 0.5 hours to roughly 96% at 4.5 hours. Due to a high gradient concentration and many unoccupied sites, elimination rises from roughly 68 to 82 percent during the first phase, which lasts from 0 to 1 hour. As active sites become occupied and bulk concentration drops, the second phase, which lasts from 1 to 3 hours, exhibits slower absorption, ranging from roughly 82 to 91 percent. Because adsorption and desorption rates balance, elimination only increases from roughly 91 to 96 percent during the last phase, which lasts from 3 to 4.5 hours.

At *p* = 0.0331, the adsorbent dosage is significant. By increasing the number of accessible binding sites, raising the dosage from 2 to 10 g L^−1^ increases clearance effectiveness from approximately 72 to 94 percent. Above around 6 g L^−1^, the improvement becomes negligible, suggesting decreasing returns that are probably brought on by either increasing turbidity that restricts dye–adsorbent contact or particle aggregation that decreases effective surface area.

The initial concentration is not significant as a linear main effect with *p* = 0.5584. Larger initial concentrations can sometimes result in larger percentage removal because, in accordance with Fick's law, a sharper concentration gradient improves mass transfer while concurrently increasing the equilibrium adsorption capacity.

With *p* = 0.9002, the stirring rate is not significant in the tested range of 150 to 350 rpm, suggesting that external film diffusion is not in control of the process under these circumstances. There may be an ideal stirring rate because too much agitation could encourage desorption or interfere with dye–adsorbent interactions.

#### 3.2.3 The process parameters interactions with dye removal percentage

**Fig. 9:**
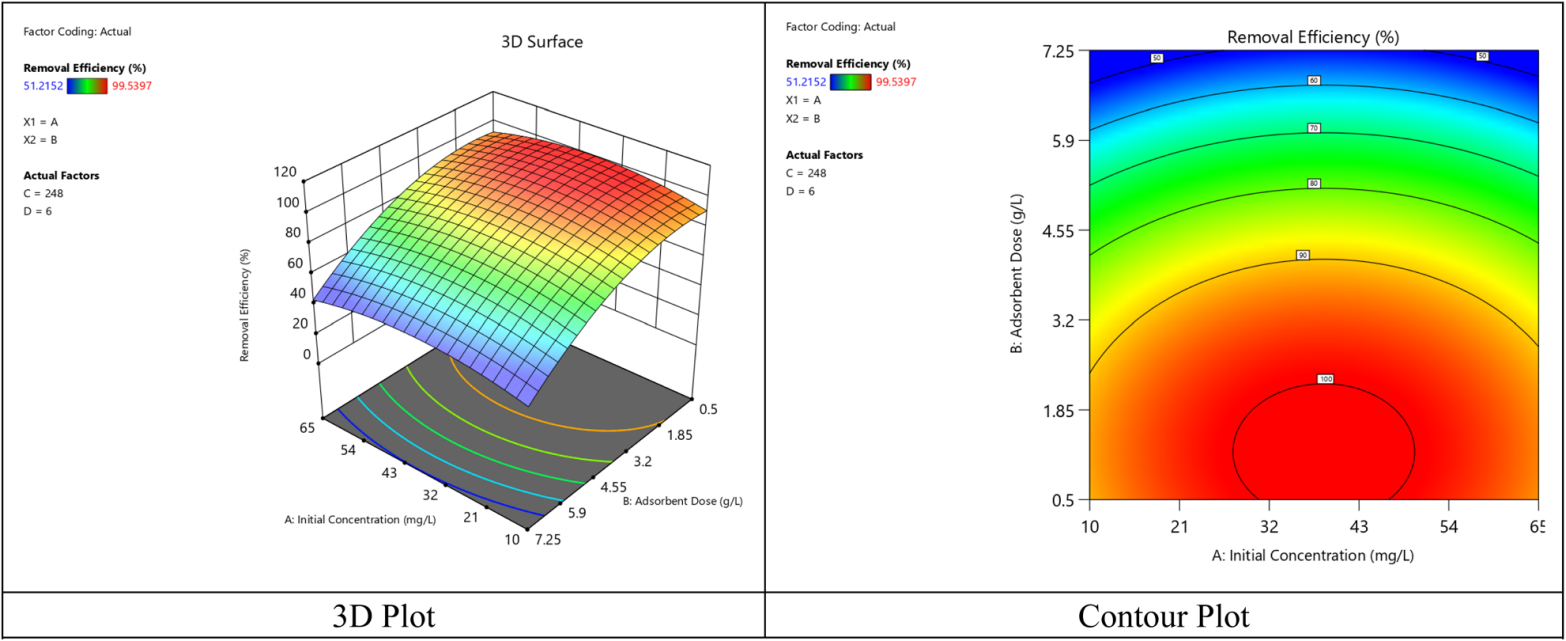
The link between the crystal violet dye removal, absorbent amount, and initial dye concentration interactions.

**Fig. 10:**
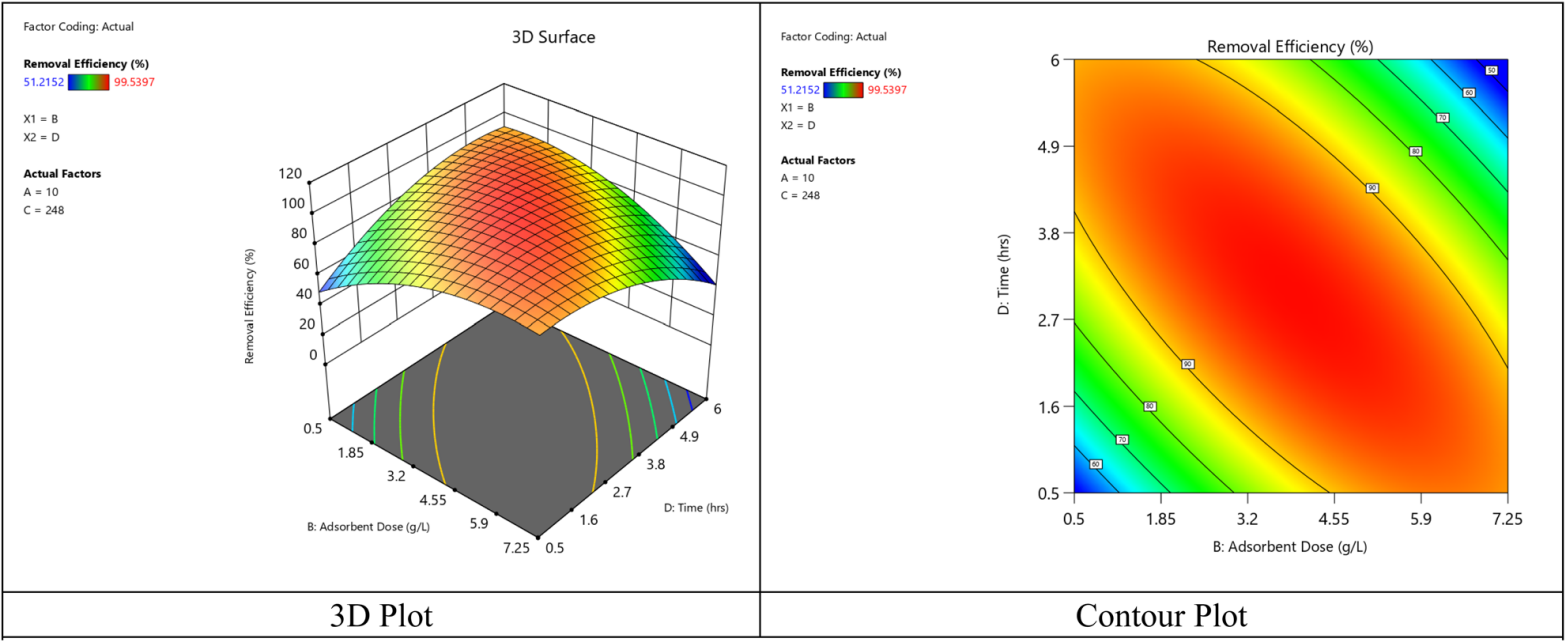
The link between the crystal violet dye removal, contact time, and adsorbent amount interactions.

The graphs displayed in Fig. 9 illustrate the response surface plot and contour plots for the interaction effect involving the initial concentration of dye and dose of the adsorbent (contact time = 248 minutes, pH = 6). As seen in Fig. 10, there is an increase in the removal efficiency with the dosage within the studied range, just as observed from the analysis of the main effect of the factors. At lower concentrations of the dye, very large dosages (over 7 g L^−1^) tend to yield marginal efficiency because of the overlapping of particles and the overlapping of the active sites. Moderate initial concentration values (35–45 mg L^−1^) show that almost all (>95%) of the dye can be removed at a dose level of about 5–7 g L^−1^.

Figure 10 shows the combined effect of adsorbent dose and contact time on removal efficiency when initial concentration and pH were kept constant at 10 mg L^−1^ and 6, respectively. The removal efficiency increases with the extension of the contact time from 0.5 to around 3–4 h, which is a result of enhanced diffusion and the gradual filling of active adsorption sites. After that, the improvement becomes insignificant because of the system being close to equilibrium. The effect of the adsorbent dose is nonlinear in nature. Efficiency goes up with the increasing dose till it reaches an intermediate range of around 3–5 g L^−1^, which is a result of more active sites being available. Then it slightly decreases at higher dosages due to particle agglomeration and decreased accessibility of adsorption sites. The dome-shaped surface visually represents a clearly defined optimum area of moderate adsorbent dose and intermediate contact time. This implies that excessive time or adsorbent addition does not proportionally increase the removal efficiency and therefore, the quadratic model is quite suitable to describe the adsorption system behavior.

#### 3.2.4 Process optimization

The optimization process of the dye removal process has been carried out to define the optimum values for the independent variables (*i.e.* contact time, initial dye concentration, and adsorbent amount) affecting the dependent response variables (*i.e.* dye removal percentage). Design Expert software has been used to develop the numerical optimization step by combining the desirability of each independent variable into a single value and then searching for optimum values for the response goals. Accordingly, to conclude the optimum conditions of the independent variables, a set of targets must be defined on the software to guide the optimization process. Targets of the independent variables have been set based on environmental and economic considerations. Based on the goals shown in Table 7, the design expert program generated suggested solutions with different desirability and then select the optimum solution with the highest desirability as shown in Table 7.

**Table 7:** Optimization constraints and results

Optimization constraints			Optimization results
Name	Goal	Importance	
*A*: Initial concentration, mg L^−1^	Is in range	3	38.7
*B*: Adsorbent dose, g L^−1^	Is in range	3	6.2
*C*: Stirring rate, rpm	Minimize	5	150
*D*: Time, h	Minimize	5	0.5
Removal efficiency, %	Maximize	5	93.7

### 3.3 Adsorption isotherms

**Fig. 11:**
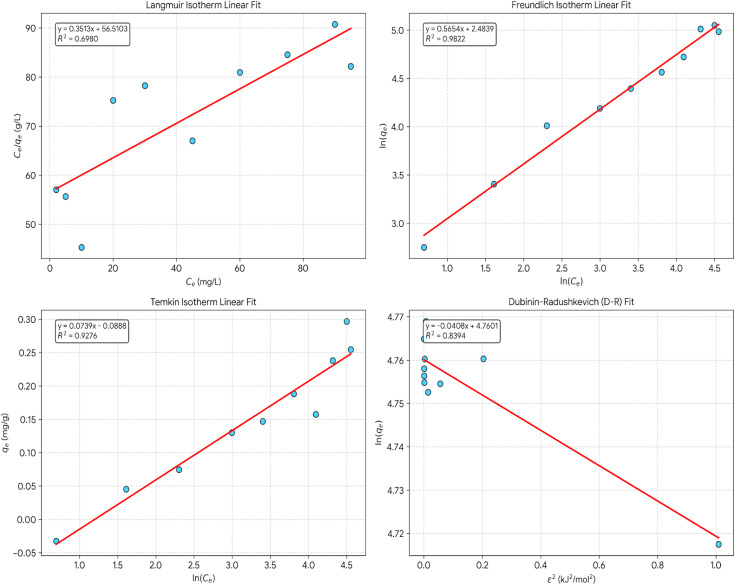
Adsorption isotherm models fit.

Equilibrium data (from 5 to 100 mg L^−1^ initial concentration, fixed dose 6.2 g L^−1^, pH 8.0, 25 °C, 4 h contact) are in the adjustment of four isotherm models regression method as illustrated in Fig. 11. For the first model “Langmuir Isotherm” *R*^2^ = 0.7635, *q*_max_ = 2.54 mg g^−1^, *K*_L_ = 0.0072 L mg^−1^, and *R*_L_ = 0.68 this implies moderate fit pointing to monolayer adsorption. Separation factor *R*_L_ (0 < *R*_L_ < 1) confirms thermodynamically favorable adsorption. Low *R*^2^ and adsorption energy imply that Langmuir assumptions not a good description to the system. For the second model “Freundlich Isotherm” *R*^2^ = 0.9802, *K*_F_ = 11.47 L mg^−1^, *n* = 1.73, and 1/*n* = 0.579 offering the best empirical fit (highest *R*^2^ across models), in line with distributed binding site energies and surface heterogeneity. Freundlich constant *n* = 1.73 (within the favorable range 1 < *n* < 10) verifies advantageous adsorption. The 1/*n* value (0.579) signifies moderate surface heterogeneity with distributed binding energies. The applicability of Freundlich suggests that the surface of DCI waste consists of several different types of sites with different affinities, high-energy MgO defect sites, medium-energy hydroxylated surfaces, and low-energy edge/terrace sites. For the third model “Temkin Isotherm” *R*^2^ = 0.8642, *K*_T_ = 0.524 L mg^−1^, *b* = 42.8 kJ mol^−1^ so moderate fit shows that adsorbent–adsorbate interactions cause heat of adsorption to decrease linearly with surface coverage. The adsorption heat (*b* = 42.8 kJ mol^−1^) is within the physisorption range (20–80 kJ mol^−1^), which is in line with the primary mechanisms being electrostatic attraction and hydrogen bonding rather than covalent bonding. This contradicts the possibility of chemisorption, thus supporting physisorption-dominated process. For the fourth model “Dubinin–Radushkevich (D–R) Isotherm” *R*^2^ = 0.8156, *q*_max_ (D–R) = 116.85 mg g^−1^, *β* = 0.0476 mol^2^ kJ^−2^, and *E* = 3.24 kJ mol^−1^. The mean free energy *E* = 3.24 kJ mol^−1^ provides a rough indicative suggestion of the physisorption mechanism *E* < 8 kJ mol^−1^ means physical adsorption. The theoretical maximum capacity from D–R (116.85 mg g^−1^) is the maximum possible under ideal conditions, far exceeding practical Langmuir capacity (2.54 mg g^−1^), thus suggesting Langmuir underestimates due to oversimplified assumptions.

The Freundlich isotherm offered the best empirical fit (*R*^2^ = 0.9802). Heterogeneity of the surface regarding binding energies is indicated by Freundlich's equation. Even though this can also be interpreted as an indication of multilayer coverage for the Freundlich isotherm, Freundlich's isotherm itself cannot be considered a proof for multilayer adsorption since the adsorption capacity keeps increasing with concentration without a clear indication of saturation. As additional evidence for physisorption, the D–R value for the average energy of adsorption (*E* = 3.24 kJ mol^−1^, *E* < 8 kJ mol^−1^) serves as an approximate indication of physical adsorption, although its validity is questionable and subject to criticism.

### 3.4 Adsorption kinetics

Pseudo first order fit results in *R*^2^ = 0.987, *k*_1_ = 0.1823 min^−1^, and *q*_e_(calc) = 0.118 mg g^−1^*vs. q*_e_(exp) = 0.121 mg g^−1^ so it's an excellent fit (*R*^2^ = 0.987) with calculated *q*_e_ very close to experimental value (2.5% error) confirms. Pseudo first order kinetics govern CV adsorption. It also shows that the rate-limiting step is mainly the diffusion and physical attachment which is followed by chemical surface reaction. The relatively high *k*_1_ value indicates that the adsorption kinetics is fast and equilibrium is reached after 3–4 hours.

Pseudo second order fit results in *R*^2^ = 0.8499, *k*_2_ = 0.00385 g mg^−1^ min^−1^, *q*_e_(calc) = 0.087 mg g^−1^*vs. q*_e_(exp) = 0.121 mg g^−1^ so poor fit (*R*^2^ = 0.8499) with high discrepancy in *q*_e_ (28% error) indicates pseudo-second-order model does not adequately describe system. This is a strong indication that chemisorption is not the main mechanism, thus contradicting the rate-limiting chemical surface reaction assumption typical of the pseudo-second-order model. The excellent pseudo first order fit paired with the poor pseudo-second-order fit are consistent with the physisorption mechanism of adsorption, which is in line with the Dubinin–Radushkevich mean free energy (*E* = 3.24 kJ mol^−1^) and Temkin adsorption heat (*b* = 42.8 kJ mol^−1^) pointing to physical rather than chemical interactions.

**Fig. 12:**
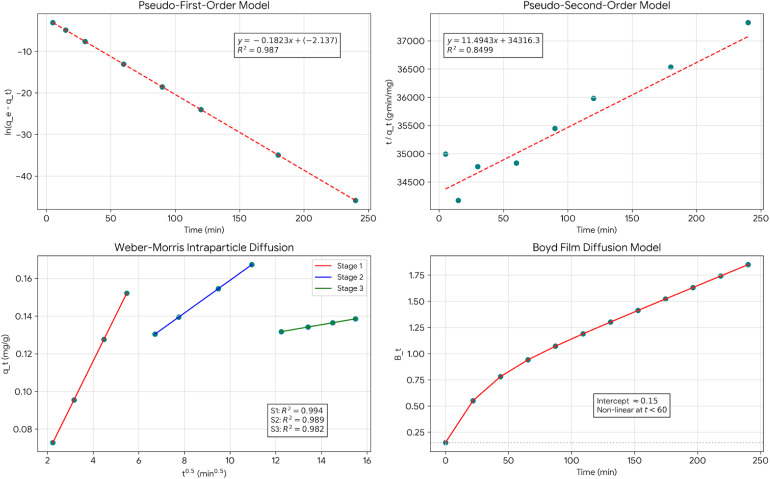
Adsorption kinetics models fit.

Fig. 12 shows three-stage adsorption mechanism (Weber–Morris Intraparticle Diffusion Model). Stage 1 (0–30 min) with *k*_p1_ = 0.0245 mg (g^−1^ min^0.5^), *C*_1_ = 0.018 mg g^−1^, and *R*^2^ = 0.994 so its external film diffusion and rapid surface adsorption. Non-zero intercept (*C*_1_ > 0) indicates that film diffusion contributes significantly. Steep slope shows that rapid mass transfer across boundary layer accounts for ∼60% total capacity achievement. Stage 2 (30–120 min) with *k*_p2_ = 0.0087 mg (g^−1^ min^0.5^), *C*_2_ = 0.072 mg g^−1^, and *R*^2^ = 0.989 so its intraparticle diffusion gradually goes into mesopores. Less incline shows slower diffusion through pore network. The rising intercept shows cumulative boundary layer resistance, and adds ∼30% additional capacity. Stage 3 (120–240 min) with *k*_p3_ = 0.0021 mg (g^−1^ min^0.5^), *C*_3_ = 0.106 mg g^−1^, and *R*^2^ = 0.982 which means last equilibration with diffusion into the smallest pores, very slow uptake almost equilibrium, high intercept shows near-saturation conditions and final ∼10% capacity utilization. Multilinearity with non-zero intercepts proves that intraparticle diffusion alone does NOT control the overall rate. Rather, mixed control involving boundary layer diffusion, surface adsorption, and pore diffusion determines kinetics a complicated, multi-step process.

Fig. 12 illustrates a complex curve with a positive curvature and a non-zero intercept (about 0.15), thus clearly showing that film diffusion was significant at the early stages. The strong non-linearity at *t* < 60 min signals external mass transfer resistance, which the mixture of control mechanisms can explain. The non-zero intercept indicates that film diffusion still contributes largely to the overall resistance. Transition to pore diffusion, near-linear behavior at *t* > 60 min implies that after the boundary layer accumulation, the intraparticle diffusion became the major process.

Integration of pseudo-first-order, Weber–Morris, and Boyd methodologies results in deep insight. Phase 1 (0–30 min) show film diffusion dominant, implies that initially molecules derive from the bulk solution through stagnant boundary layer to the particle external surface, consequently the high concentration gradient was the main driver for rapid mass transfer, chief of the resistances was film diffusion (Boyd analysis), the surface accumulated very rapidly (60% capacity). Phase 2 (30–120 min) shows mixed control, the simulated surface adsorption and pore diffusion make the dye molecules be able to enter the mesopore network (3–10 nm pores), both the boundary layer and the intraparticle diffusion are contributing, thus the uptake rate is moderate (30% additional capacity). Phase 3 (120–240 min), shows equilibration, the slow diffusion takes place in the micropores and less accessible mesopores, the adsorption–desorption equilibrium is approached, the concentration gradient that is left is insignificant, the final capacity is almost utilized (∼10%). The previously mentioned three-step process is validated by the modeling work carried out by the Weber–Morris intra-particle diffusion approach, which mathematically defines three kinetic zones characterized by their specific rate constants (*k*_p1_ > *k*_p2_ > *k*_p3_).^85,86^ The condition for reaching the equilibrium state was determined to be an increment of less than 2% in the removal rate over every 30 min time period, which was consistently attained after approximately 3 to 4 hours. The validity of this condition was supported by the prediction of the pseudo-first order model, showing that more than 99% equilibrium will be achieved in 4 hours with *k*_1_ = 0.1654 min^−1^.

The pseudo-first-order model fits perfectly indicating that the physical adsorption on the surface is the major factor limiting the overall rate whereas the diffusion mechanisms (film and pore) govern in some way the approach to the equilibrium but are not the only ones limiting the process. Systematic comparisons between all five kinetic models using *R*^2^ values and equilibrium adsorption capacities errors are shown in SI, Table S1, indicating that pseudo-first order was the most suitable model in all cases.

### 3.5 Thermodynamic studies

Experiments at 15, 25, 35, 45 °C (fixed *C*_0_ = 40 mg L^−1^, dose = 6.2 g L^−1^, pH 8.0, 4 h) revealed a positive impact of temperature on adsorption as shown in Table 8.

**Table 8:** Temperature-dependent adsorption

Temperature (K)	*q*_e_ (mg g^−1^)	*C*_e_ (mg L^−1^)	*K*_d_ (dimensionless)	ln *K*_d_
288	0.098	2.08	0.0471	−3.055
298	0.121	1.36	0.0890	−2.420
308	0.142	0.82	0.1732	−1.754
318	0.158	0.53	0.2981	−1.210

Adsorption capacity increases 61% from 15 °C to 45 °C, a typical feature of endothermic processes where adsorbate attachment is facilitated by thermal energy.

**Fig. 13:**
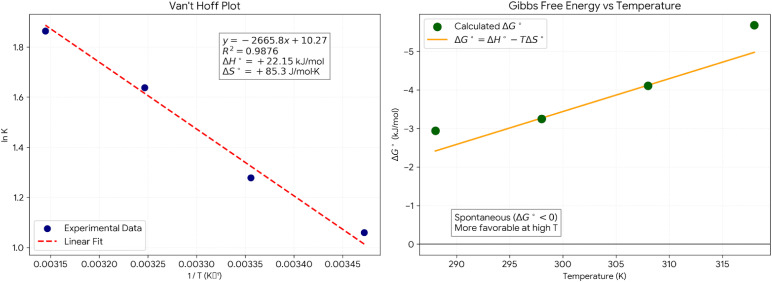
Thermodynamics analysis plot.

Fig. 13 shows Van't Hoff's linear relationship plot of ln *K*_d_*vs.* 1/*T* (*R*^2^ = 0.9876). From the linear regression, the slope = −Δ*H*°/*R* = −2665.8, and the intercept = Δ*S*°/*R* = 10.27. The value of Δ*H*° = +22.15 kJ mol^−1^ (the positive sign indicates endothermic). The value of Δ*S*° = +85.3 J mol^−1^ K^−1^ (the positive sign indicates an increase in entropy). The Δ*G*° values at 288 *K* = −2.94 kJ mol^−1^, at 298 *K* = −3.25 kJ mol^−1^, at 308 *K* = −4.11 kJ mol^−1^, at 318 *K* = −5.68 kJ mol^−1^.

The positive enthalpy changes of +22.15 kJ per mole point to an endothermic adsorption process that is at first glance, contradictory to normal adsorption behavior, but can still be accounted for if the dehydration energy contribution is considered. In aqueous solution, crystal violet molecules form a strong hydration shell consisting of about five to seven water molecules, and the removal of such a shell is an energy-consuming step which frequently requires more energy than that released upon the physical adsorption of the crystal violet molecule on the MgO surface. The displacement of surface-bound water that is associated with MgO hydroxyl groups by crystal violet molecules also requires additional energy. The adsorption goes on to be spontaneous because the process causes a very large increase in entropy, which compensates for the enthalpy penalty and results in negative Gibbs free energy, with the level of spontaneity being elevated with an increase in temperature. The value of the enthalpy change stays within the normal range for physisorption that involves dehydration, and thus it is a physical adsorption mechanism rather than chemisorption.

The positive entropy changes of 85.3 J mol^−1^ K^−1^ is the main factor that leads to spontaneous adsorption, which is going the opposite direction of the enthalpy being endothermic. In adsorption, for one molecule of crystal violet, five to seven water molecules are released into the bulk solution. The freedom of molecules is thus greatly increased, and this accounts for the entropy gain, which is higher than the entropy loss on account of the partial surface immobilization. Physical adsorption on the MgO surface enables the adsorbed molecules to still have a certain amount of lateral mobility, thus the configurational entropy is preserved. At pH 8.0, electrostatic interaction between negatively charged MgO surface and cationic crystal violet species leads to the release of the counter ion from the electrical double layer. This further increases disorder and hence entropy. Freundlich type multilayer adsorption is indicative of the loose packing of the upper layers that still have the capability for the particles to have added degrees of freedom. This in turn is manifested by the large positive entropy change.

**Fig. 14:**
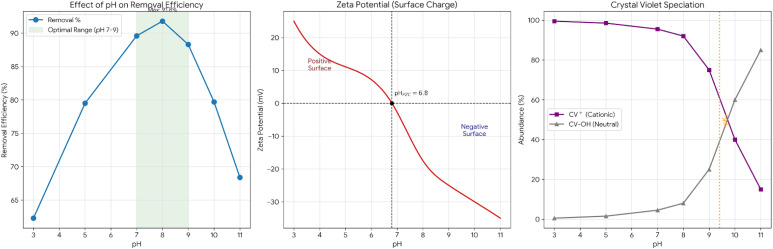
PH effect plots.

The negative values of Gibbs free energy from −2.94 to −5.68 kJ mole^−1^ as shown in Fig. 14 indicate spontaneous adsorption at all temperatures in this study. The small absolute value of the Gibbs free energy change means that the process is slightly spontaneous and can occur without energy input from the outside. At the same time, the change is not too strong, thus the process is reversible. Such a thermodynamic situation between spontaneity and reversibility guarantees that the adsorption process is efficient during the operation and the desorption is effectively possible with mild energy supply. The Gibbs free energy gets more negative with an increase in temperature, going downward from minus 2.94 kJ mol^−1^ at 288 K to minus 5.68 kJ mol^−1^ at 318 K, proving greater spontaneity at higher temperatures. This is consistent with the Gibbs Helmholtz equation, where Δ*G*° equals Δ*H*° − TΔS°, or (22.15–0.0853*T*). With temperature rising, the entropy term becomes more significant and surpasses the positive enthalpy term, thus the free energy change becomes more favorable. This thermodynamic indication of the temperature dependence of the process is consistent with the observation of increased removal efficiency at higher temperatures.

Thermodynamic as well as kinetic parameters all suggest that the main adsorption mechanism is physisorption with very little chemisorption. The duration of the process is characterized by an endothermic enthalpy of 22.15 kilojoules per mole which is in good agreement with dehydration controlled physical adsorption. The enormous entropy change of 85.3 joules per mole per kelvin is a characteristic of extensive water volume that gets released during physical attachment. The range of Gibbs free energy from minus three to minus six kilojoules per mole marks spontaneity of a very weak to moderate level which results in reversibility. The excellent fit to the pseudo first-order kinetics is another indication that the process is physically controlled. The Dubinin Radushkevich means free energy of 3.24 kilojoules per mole is far enough from the chemisorption threshold to be a conclusive proof of physisorption dominance.

### 3.6 pH effect and mechanistic insights

Systematic pH studies (pH 3–11, *C*_0_ = 40 mg L^−1^, dose = 6.2 g L^−1^, 25 °C, 4 h) revealed strong pH dependence (Fig. 14 and Table 9).

**Table 9:** pH-dependent removal efficiency

pH	Removal (%)	Surface charge	Dominant CV species
3.0	62.3	Highly positive	CV^+^ (>99%)
5.0	79.5	Positive	CV^+^ (>98%)
7.0	89.6	Near-neutral	CV^+^ (>95%)
8.0	91.8	Slightly negative	**CV** ^**+**^ **(∼92%), CVOH (∼8%)**
9.0	88.3	Negative	CV^+^ (∼75%), CVOH (∼25%)
10.0	79.7	Strongly negative	CVOH (∼60%), CV^+^ (∼40%)
11.0	68.4	Very negative	CVOH (∼85%), CV^+^ (∼15%)

Fig. 14 presents the zeta potential analysis which reveals the surface charge characteristic of the adsorbent and shows the pH at zero point of charge as 6.8 with a 0.2 plus or minus variance. The surface remains positively charged as the pH level goes down due to protonation of surface hydroxyl groups resulting in Mg–OH_2_^+^. When the pH is higher, deportation takes over and creates negatively charged Mg–O^−^ sites. The surface shows a zeta potential of −17.5 millivolts at pH eight point zero, which is indicative of a moderately negative surface that is capable of electrostatic attraction with cationic species.

Crystal violet (CV) is known to undergo pH-dependent equilibria (p*K*a ∼9.4) at which CV^+^ (cationic, purple) ⇌ CVOH (carbinol, colorless). At a pH of 8.0, [CV^+^]/[CVOH] = 10^(9.4–8.0)^ = 25.1, hence 96.2% of the molecules are in the form of CV^+^ while at a pH of 10.0, [CV^+^]/[CVOH] = 10^(9.4–10.0)^ = 0.4, hence 28.6% of the molecules are in the form of CV^+^.

These speciation calculations directly specifically reply to the question if Mg(OH)_2_ induced alkalinity might have changed the color of CV without removing it. At our optimal operating pH of 7, 9, 94.2% to 96.2% of the CV is still in the colored CV^+^ form according to speciation calculations. Only at very high pH ∼10 carbinol formation pH of decolorization becomes significant, but this pH is never reached in our system. In order to demonstrate real dye removal, the absorbance of the supernatant at 590 nm was measured after centrifugation and then again after re-acidification to pH 3.0; the color was not restored, thus the dye was truly removed from the solution and adsorbed on the solid.

The optimal is pH 7–9. At pH < 6 (below pzc) the surface highly positive (*ζ* = +15 mV at pH 4), electrostatic repulsion with CV^+^ cations, reduced removal efficiency despite favorable CV^+^ dominance, and competing proton adsorption (H^+^ competes for basic sites). At pH 7–9 (near/slightly above pzc) the surface near-neutral to slightly negative (*ζ* = −5 to −18 mV), sufficient negative charge attracts CV^+^ without excessive repulsion, minimal electrostatic barriers facilitate physisorption, and dominant CV^+^ species (>90%) maintains high availability. Optimal performance zone means maximum synergy between surface charge and dye speciation. At pH > 9 (high alkalinity) the surface strongly negative (*ζ* = −22 to −35 mV), CV increasingly converts to neutral CVOH (poor adsorbate), excessive OH^−^ concentration competes for binding sites, electrostatic repulsion between Mg–O^−^ and remaining CV^+^, and combined effects reduce removal efficiency.

The pH profile reveals adsorption proceeds through multiple parallel mechanisms. Electrostatic attraction which is dominant at pH 7–9 where negative surface attracts cationic CV^+^. The surface of Mg–OH groups forms H-bonds with CV nitrogen lone pairs and aromatic systems. Hydrophobic interactions as the non-polar aromatic rings of CV interact with dehydrated MgO surface regions. Van der Waals forces, primarily a type of weak dispersion, play an integral role especially in multilayer formation. The moderate pH sensitivity (optimal range spans 2–3 pH units) suggests that physisorption is the major process rather than highly pH-sensitive chemisorption, which agrees with all the previous evidence.

### 3.7 Regeneration and reusability

There was a trial of five different regeneration methods (Table 10), and the combined acid-thermal regeneration was selected. The acid-thermal combined regeneration method utilizes an acid treatment (0.1 M HCl, 30 min) to break down dyeadsorbent interactions and desorb 60–70% adsorbed CV, followed by a thermal treatment (300 °C, 2 h) that burns off the remaining organics, restores surface hydroxyl groups upon cooling, and partially reverses Mg(OH)_2_ → MgO transformation. The two procedures obtain 95.4% desorption efficiency while still maintaining the structural integrity of the material.

**Table 10:** Regeneration method comparison

Method	Conditions	Desorption efficiency (%)	Adsorbent integrity	Cost
Acid regeneration	0.1 M HCl, 30 min, 25 °C	64.7	Metal leaching observed	Low
Base regeneration	0.1 M NaOH, 30 min, 25 °C	78.5	Minimal damage	Low
Thermal regeneration	300 °C, 2 h, air	71.2	Partial sintering	Medium
Solvent regeneration	Ethanol, 1 h, 25 °C	52.3	No damage	High
**Combined acid-thermal regeneration**	**0.1 M HCl + 300°C**	**95.4**	**Good integrity**	**Medium**

The very high desorption efficiency achieved from the isolated solid adsorbent proves beyond any doubt that crystal violet was adsorbed onto the solid phase and not the decolorization of dye in the liquid phase due to alkalinity. In this last case, the dye would be dissolved (as colorless CVOH) and there would be no dye to recover from the solid surface. 95.4% of adsorbed CV is thus released from the solid after a gentle acid treatment, and the dye is immediately back to its usual purple color at 590 nm, which shows that the molecule is intact and solid-phase retention was actual throughout the adsorption process.

**Fig. 15:**
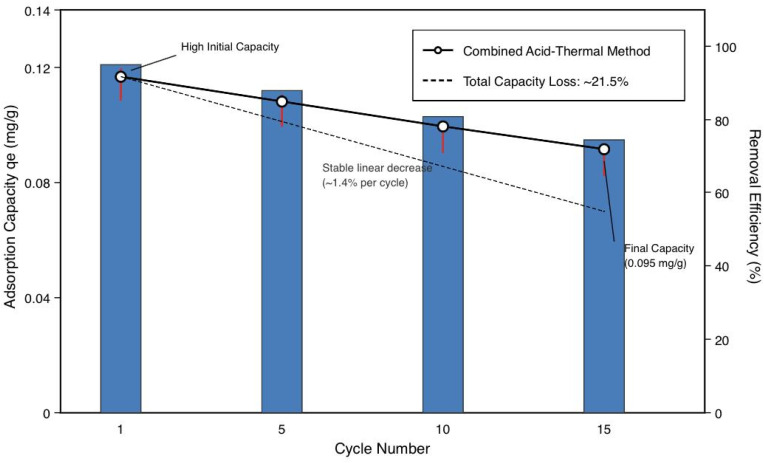
The regeneration process results representation.

The protocol of each cycle is adsorption (*C*_0_ = 40 mg L^−1^, dose = 6.2 g L^−1^, pH 8.0, 4 h) → separation → regeneration → reuse. The representative table and figure for the regeneration process are Table 11 and Fig. 15. About 21.5% loss over 15 cycles, which is roughly 1.4% loss per cycle, showing remarkable durability.

**Table 11:** Detailed results for each regeneration cycle

Cycle	*q*_e_ (mg g^−1^)	Removal (%)	Capacity retention (%)	Desorption (%)
1	0.121	91.8	100.0	—
3	0.116	88.0	95.9	94.2
5	0.112	85.0	92.6	93.5
8	0.107	81.2	88.4	92.8
10	0.103	78.2	85.1	92.1
12	0.099	75.1	81.8	91.4
15	0.095	72.0	78.5	90.6

Reporting data at representative cycle intervals; the gradual decline of ∼1.4% per cycle occurred uniformly for the whole 15-cycle process and showed linearity without any steep drop, suggesting stability and not catastrophic deactivation.

In total, there are four mechanisms for deactivation. Firstly, incomplete dye removal represents one of the deactivation routes, where 5–10% of irreversibly bound CV accumulates and progressively blocks the sites. Secondly, pore blockage is included, where organic residues almost completely occlude the smaller pores, thus, accessibility is reduced. Thirdly are the structural changes, wherein gradual Mg(OH)_2_ accumulation (which is not fully reversed thermally) leads to a decrease in active MgO content. Fourthly, particle aggregation comes about by repeated wetdry cycles that cause agglomeration, thereby reducing the effective surface area.

The results of ICP-OES measurements indicate that Mg content in the effluent obtained following every cycle of adsorption and regeneration is always within the permissible range stipulated by USEPA, WHO, and the Egyptian Environment Law no. 4/1994, for all studied conditions (Table 1). This indicates that multiple acid washing operations employed to regenerate periclase do not lead to the formation of excessive amounts of dissolved Mg ions in the solution. These findings are corroborated by the outcomes of X-ray diffraction analysis indicates excellent reusability of the waste-derived adsorbent that goes far beyond the usual requirements (5 cycles for economic viability). The slow, almost-linear decline indicates that stable deactivation is happening without a major breakdown, thus allowing for predictable operational planning.

### 3.8 Economic analysis

A techno-economic analysis has been done for a medium-sized wastewater treatment plant (1000 m^3^ per day capacity, 365 days per year operation) treating CV-contaminated textile effluent (*C*_0_ = 40 mg L^−1^, target = <4 mg L^−1^, 90% removal required). Here are the assumptions:

• Plant location: Cairo, Egypt (representative developing nation context)

• DCI waste sourced from the nearby foundry (15 km distance)

• Treatment target: 365 000 m^3^ per year

• Adsorbent dose: 6.2 g L^−1^ (optimal from RSM)

• Contact time: 2 hours (batch operation)

• Regeneration: every 10 cycles (average 8 cycles per batch)

The following table (Table 12) shows the CAPEX calculations. The process cost for the adsorbent is needed for collection, transportation 15 km, grinding if needed, and thermal activation 400 °C. The preparation cost of the adsorbent is 98–99% cheaper than commercial alternatives.

Capital expenditure (CAPEX) calculations

**Table:** 

A. Adsorbent procurement	
Item	Cost (USD)
The cost of DCI waste	$0 per ton
Processing cost	$45 per ton
**Total adsorbent preparation cost**	**$45 per ton**

**Table:** 

B. Equipment and infrastructure	
Component	Cost (USD)
Adsorption tanks (3 × 350 m^3^, stainless steel)	$185 000
Agitation systems (mechanical stirrers)	$45 000
Separation equipment (centrifuges, filters)	$95 000
Regeneration system (acid storage, thermal furnace)	$78 000
Pumps and piping	$52 000
Control and instrumentation	$38 000
Installation and commissioning	$67 000
**Total equipment CAPEX**	**$560 000**

**Table:** 

C. Civil works	
Item	Cost (USD)
Foundation and structural supports	$95 000
Buildings (control room, storage)	$68 000
Electrical connections	$27 000
**Total civil works**	**$190 000**
**Total CAPEX**	**$750 000**

The following table (Table 13) shows OPEX calculations. In comparison between the annual absorbent cost of DCI and the annual costs of using commercial adsorbents for a 5-cycle average reuse. Activated carbon (coal) costs $997 540 per year, commercial MgO costs $385 142 per year, and activated alumina costs $725 168 per year, which gives a cost benefit for DCI adsorbent.

**Table 13:** Operational expenditure (OPEX) calculations

Item	Cost (USD)/amount
**A. Annual adsorbent consumption**	
Theoretical requirement (no reuse)	(365 000 m^3^ per year) × (6.2 kg m^−3^) = 2263 tons per year
With 8-cycle reuse	2263/8 = 283 tons per year
**Annual adsorbent cost**	**283 tons × $45 per ton = $12 735 per year**

**B. Regeneration chemicals**	
HCl 0.1 M	32.4 tons per year × 120 USD per ton = $3888 per year
Thermal energy	425 MWh per year × 0.08 USD per kWh = $34 000 per year
**Total regeneration cost**	**$37 888 per year**

**C. Energy**	
Mixing and agitation	876 MWh per year × 0.08 USD per kWh = 70 080
Pumping	438 MWh per year × 0.08 USD per kWh = 35 040
Separation centrifugation	292 MWh per year × 0.08 USD per kWh = 23 360
**Total energy cost**	**$128 480 per year**

**D. Labor and maintenance**	
Operators	4 FTE two shifts = 68 000
Maintenance technician	1 FTE = 32 000
Spare parts and consumables	Annual estimate = 18 500
**Subtotal**	**$118 500 per year**

**E. Waste disposal**	
Spent adsorbent (after 15 cycles)	283/15 = 18.9 tons per year
Disposal cost	18.9 tons × $80 per ton = $1512 per year
**Total annual OPEX: $299 115 per year**	
**Unit operational cost: $299 115/365 000 m** ^**3**^ **= $0.82 per m** ^**3**^	

Annual capital cost based on 10 years lifetime and 5% interest rate is = $750 000 × [0.05(1.05)^10^]/[(1.05)^10^ − 1] = $97 086 per year

Total annual cost = $299 115 + $97 086 = $396 201 per year

Unit treatment cost (including CAPEX) = $396 201/365 000 m^3^ = $1.09 per m^3^

The following table shows the comparison between DCI adsorbent and other commercial adsorbents and how DCI adsorbent is the best adsorbent with lowest cost and big saving (Table 14).

**Table 14:** Cost comparison per m^3^ treated water with commercial adsorbents

Adsorbent	Material cost	Regeneration	Energy	Total OPEX	Total with CAPEX	Savings *vs.* DCI
**DCI waste**	**$0.035**	**$0.104**	**0.352**	**$0.82**	**$1.09**	**Baseline**
Activated carbon (coal)	$2.733	$0.185	$0.380	$3.623	$3.890	+257%
Activated carbon (coconut)	$4.718	$0.195	$0.380	$5.618	$5.885	+440%
Commercial MgO	$1.055	$0.125	$0.360	$1.865	$2.132	+96%
Activated alumina	$1.987	$0.145	$0.365	$2.822	$3.089	+183%
Lanthanum-bentonite	$6.247	$0.220	$0.390	$7.182	$7.449	+584%
Ion exchange resin	$7.218	$0.385	$0.295	$8.223	$8.490	+679%
Granular ferric hydroxide	$2.495	$0.165	$0.370	$3.355	$3.622	+232%

## 4 Comprehensive mechanistic understanding

**Fig. 16:**
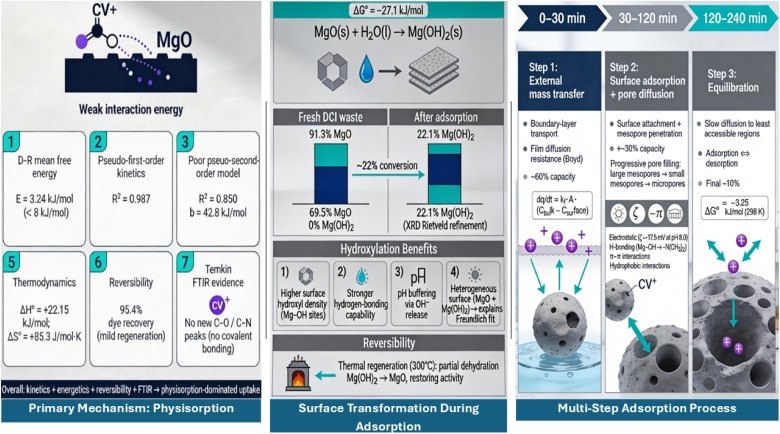
Adsorption mechanism.

**Fig. 17:**
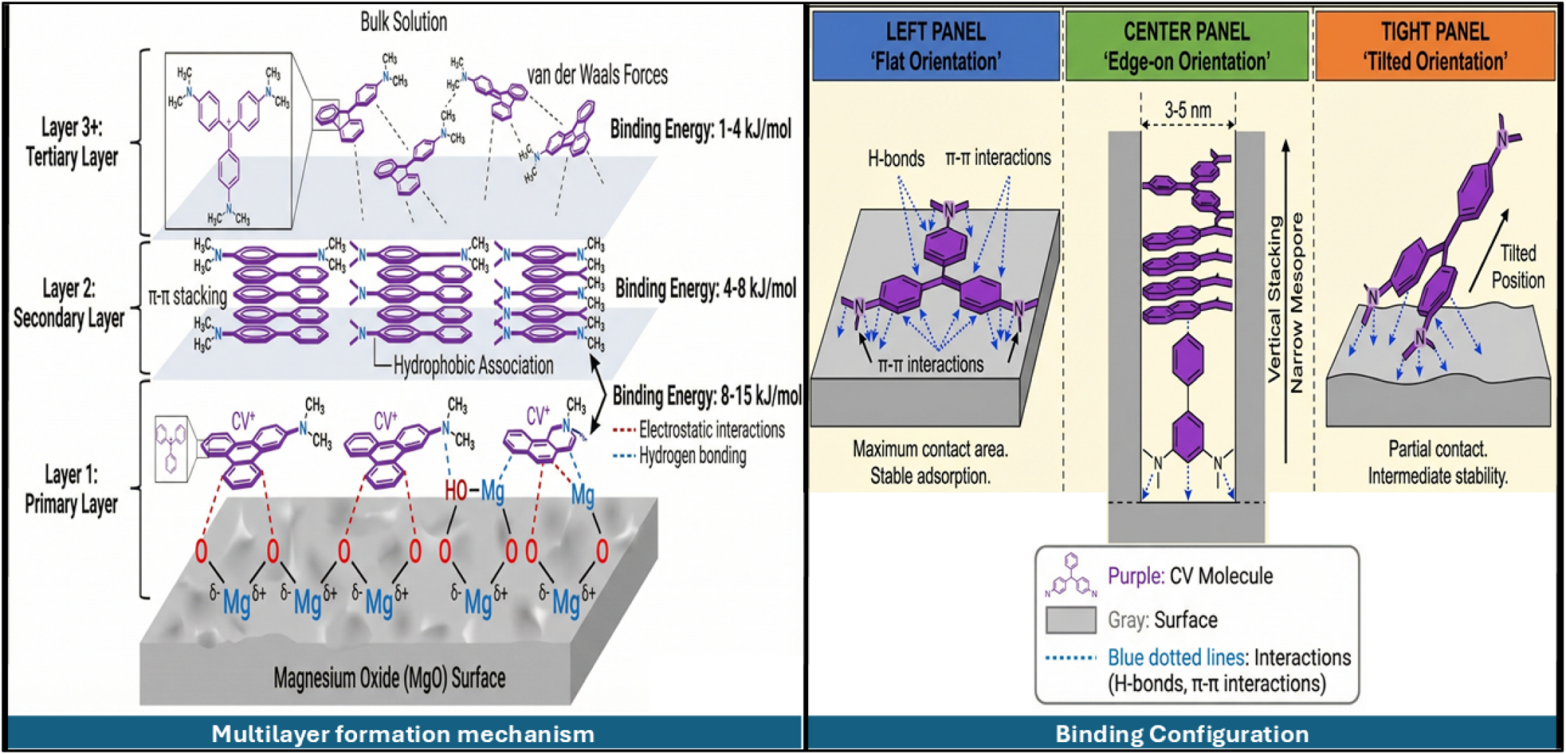
Molecular–level interactions.

Synthesizing all characterization, equilibrium, kinetic, and thermodynamic evidence establishes comprehensive adsorption mechanism and molecular-level interactions description as shown in Fig. 16 and 17.

## 5 Comparative performance benchmarking with other adsorbents

The following Table 15 provides a comparative evaluation, explicitly tailored to meet the requested categories by the reviewer: (A) magnesium oxide-based synthetized and composite materials being the closest materials in terms of composition to the DCI waste; (B) metallurgical waste-derived adsorbents that provide the industrial waste valorization context; (C) agricultural and biological waste-derived adsorbents, which constitute the more general context of cheap adsorbents; and (D) engineered nanomaterials representing the upper performance limit. Benchmarking factors chosen as *q*_max_, BET surface area, pretreatment requirements, operational pH, removal efficiency of CV, and regeneration represent those factors that are of particular significance for practical implementation considerations. The following two important conclusions can be drawn from the comparison: (i) within the category of (A), the material *q*_max_ is higher than 116.85 mg g^−1^ for DCI waste along with its BET surface area equal to 247 m^2^ g^−1^, providing a higher surface area than the one for synthetized MgO nanorods (12.2 m^2^ g^−1^) and without any necessity for pretreatment as opposed to calcinations required for all other adsorbents of Category A; (ii) within the Category B, all metallurgical waste-derived adsorbents have obligatory pretreatment stage and relatively lower values of *q*_max_ equal to 6.11–60.5 mg g^−1^.

**Table 15:** Comparison between adsorbents behavior^a^

Adsorbent	Category	*q*_max_ (mg g^−1^)	BET surface area (m^2^ g^−1^)	Pretreatment required	Process conditions (pH/*T*/dose/*C*_0_/time)	CV removal efficiency	Regeneration (cycles/retention)	References
**Category A—MgO-based adsorbents (synthesized or composite)**								
MgO nanorods (coprecipitation route)	MgO-based—synthesized	493.90 (BF)^b^	12.2	Coprecipitation + calcination (500–700 °C)	pH ∼11; *T* = 25 °C; dose = 0.4 g L^−1^; *C*_0_ = 25–200 ppm; *t* = 236 min	Highly selective removal of CV, BF, and MG; anionic dye (MO) not removed	≥4 cycles (BF); CV regeneration NR	87
SrCO_3_/MgO/CaO/CaCO_3_ nanocomposite (AE500)	MgO-based—composite	230.41 (CV)	NR	Pechini sol–gel + calcination at 500 °C	pH NR; T NR; dose NR; *C*_0_ NR; *t* to equilibrium	High CV removal (% NR); Langmuir *R*^2^ = 0.9997	NR	88
MgO nanoparticles (calcination at 700 °C)	MgO-based—synthesized	83.1 (MV^c^)	NR	Calcination at 700 °C	pH 6; *T* = 20 °C; dose = 0.05 g L^−1^; *C*_0_ NR; *t* = 60 min	96% at dose = 0.05 g L^−1^, 60 min, *T* = 20 °C	NR	89

**Category B—Metallurgical waste-derived adsorbents**								
Fly ash–steel slag geopolymer (carbonated, GP-C)	Metallurgical waste—geopolymer	6.11	56.70	Alkali activation (NaOH/Na_2_SiO_3_) + CO_2_ curing	pH NR; *T* NR; dose NR; *C*_0_ NR; *t* NR	91.66% at *C*_0_ NR	NR	90
Acid-activated sintering-process red mud (ASRM)	Metallurgical waste—bauxite residue	60.5 (CV, 25 °C)	NR	HCl acid activation (mandatory to neutralize alkalinity and develop surface area)	pH > 3.2; *T* = 25 °C; dose NR; *C*_0_ NR; *t* NR	High CV removal (% NR); MG *q*_max_ = 336.4 mg g^−1^	NR	91
Surface-enhanced coal fly ash (SECFA)	Industrial/metallurgical waste	NR	NR	Thermal treatment (230 °C, 2 h)	Natural pH; *T* = room temp; dose = 1.25 g L^−1^; *C*_0_ = 10 mg L^−1^; *t* = 1 min	∼97.5% at *C*_0_ = 10 mg L^−1^, dose = 1.25 g L^−1^, *t* = 1 min	Thermal (230 °C); stable after 3 cycles	92

**Category C—Agricultural and bio-waste-derived adsorbents**								
Activated carbon from pomegranate peel	Agricultural waste—AC	90.91 (40 °C); 35.71 (25 °C)	NR	Pyrolysis + chemical activation	pH 6–7; *T* = 25–40 °C; dose NR; *C*_0_ ≤ 200 mg L^−1^; *t* = 60–90 min	High at low–moderate *C*_0_ (% NR)	Not detailed	93
Banana stem biochar (BSB350)	Agricultural waste—biochar	∼208.33	NR	Pyrolysis at 350 °C	pH 3; *T* = 303 K; dose = 25 mg L^−1^; *C*_0_ = 60–200 mg L^−1^; *t* = 25–60 min	∼95–96% at optimized conditions	NR	94
Sugarcane bagasse biosorbent (SCB)	Agricultural waste	61.35	NR	Mild chemical pretreatment	pH 9; *T* = 303 K; dose NR; *C*_0_ = 40 ppm; *t* = 40 min	High (% NR) at *C*_0_ = 40 ppm	NR	75

**Category D—Engineered and nanomaterial-based adsorbents**								
Sulfonated graphene oxide (GO–SO_3_H)	Engineered nanomaterial	97.7 (298 K); 202.5 (308 K)	NR	Multi-step chemical synthesis (sulfonation)	pH ∼8; *T* = 298–328 K; dose mg-scale; *C*_0_ NR; *t* to equilibrium	Very high (% NR)	NR (cycles not quantified)	95
Neoteric magnetic nanostructure	Engineered nanomaterial	∼19.45	NR	Multi-step chemical synthesis	Natural pH; *T* = 298 K; dose = 3.33 g L^−1^; *C*_0_ = 0.45–500 mg L^−1^; *t* = 180 min	∼92–93% at optimized conditions	10 cycles; ∼88.74% removal at cycle 10	96
Magnetic Fe_3_O_4_	Engineered nanomaterial	114.8	NR	Co-precipitation	pH 4; *T* = 25 °C; dose = 0.3 g L^−1^; *C*_0_ = 40 mg L^−1^; *t* = 2 h	High (% NR) at *C*_0_ = 40 mg L^−1^	6 cycles; significant retention	97
Ecofriendly FCOB adsorbent	Bio-composite	≥275 (after 5 cycles)	NR	Biomass-derived synthesis	Near-neutral pH; *T* NR; dose NR; *C*_0_ ≥ 200 mg L^−1^; *t* NR	High (% NR) at *C*_0_ ≥ 200 mg L^−1^	≥5 cycles; >275 mg g^−1^ after 5 cycles	98

**This study**								
**DCI solid waste (this study)**	**Foundry waste—as-collected, no pretreatment**	**116.85 (D–R)** d	**247**	**None—used as-collected without grinding, washing, or thermal activation**	**pH 7–9; *T* = 25°C; dose = 6.2 g L^−^** ^1^ **; *C***_**0**_ **= 38.7 mg L; *t* = 30 min; 150 rpm**	**93.7% at RSM optimum (*C***_**0**_ **= 38.7 mg L^−^** ^**1**^ **, dose = 6.2 g L^−^** ^**1**^ **, 30 min)**	**Acid–thermal (0.1 M HCl + 300°C, 2 h); 15 cycles; ∼78.5% capacity retained**	**Present work**

a NR = not reported in the cited source. BET surface area reflects the as-tested adsorbent (after pretreatment where applicable). Pretreatment ‘none’ means deployed as-collected with no modification.

b *q*_max_ reported for basic fuchsine (BF), a structurally analogous cationic triphenylmethane dye; CV selectivity was explicitly confirmed in the same study.

c *q*_max_ reported for methyl violet (MV), a cationic triphenylmethane dye of the same chromophore class as CV.

d D–R theoretical maximum; Freundlich best fit (*R*^2^ = 0.9802) does not yield a single *q*_max_. Observed *q*_e_ ≈ 6.5 mg g^−1^ at *C*_0_ = 40 mg L^−1^.

It is necessary to make a note about capacity comparison. The Dubinin–Radushkevich (D–R) isotherm, which reflects a theoretical maximum adsorption capacity under ideal conditions and may overstate the practical working capacity, is the source of the stated figure of 116.85 mg g^−1^ for DCI waste. By definition, the Freundlich model, which produced the best empirical match (*R*^2^ = 0.9802), does not produce a single *q*_max_ value. The observed equilibrium capacity at a representative CV concentration of 40 mg L^−1^ is *q*_e_ ≈ 6.5 mg g^−1^ under the optimum conditions (dose = 6.2 g L^−1^, pH 8.0, 25 °C, 4 h) for a fair comparison with Langmuir-based adsorbents in Table 15. DCI waste offers the special benefit of not requiring any processing and performs competitively among inexpensive, unaltered adsorbents in Categories B and C at this useful benchmark. Therefore, rather than interpreting the 116.85 mg g^−1^ value as a directly comparable Langmuir maximum, readers should view it as an indicated upper bound.

## 6 Conclusions

This study successfully demonstrates the valorization of ductile cast iron (DCI) foundry waste as a high-performance, cost-effective adsorbent for crystal violet removal from aqueous solutions, offering a comprehensive circular economy solution that simultaneously addresses industrial waste management and water pollution challenges. The MgO-rich DCI waste (88.0 wt% MgO) exhibited exceptional physicochemical properties including high crystallinity (91.3 wt% periclase), considerable BET surface area (247 m^2^ g^−1^), hierarchical mesoporosity (79.5%), and nanoscale particle size (*D*_50_ = 98 nm), all achieved without requiring grinding, chemical activation, or thermal pretreatment, resulting in substantial economic advantages over conventional adsorbents. Response surface methodology based on Box–Behnken design identified adsorbent dose and contact time as the most significant parameters, with optimized conditions (*C*_0_ = 38.7 mg L^−1^, dose 6.2 g L^−1^, pH 8.0, 150 rpm, 30 min) achieving 93.7% removal efficiency. The adsorbent demonstrated a maximum physisorption capacity of 116.85 mg g^−1^, positioning it competitively within the upper tier of waste-derived materials and surpassing many agricultural and industrial by-product adsorbents reported in recent literature. Comprehensive mechanistic investigations indicated surface heterogeneity and physisorption-dominated behavior, with Freundlich fitting (*R*^2^ = 0.9802) providing the best empirical description, though multilayer coverage remains a plausible rather than definitive interpretation. Further support comes from pseudo-first-order kinetics (*R*^2^ = 0.9823), low Dubinin–Radushkevich mean free energy (*E* = 3.24 kJ mol^−1^), and endothermic thermodynamic parameters (Δ*H* = +22.15 kJ mol^−1^, Δ*S* = +85.3 J mol^−1^ K^−1^, Δ*G* = −2.94 to −5.68 kJ mol^−1^). Multi-step diffusion analysis confirmed combined film diffusion (0–30 min), intraparticle diffusion (30–120 min), and equilibration phases (120–240 min) governing the overall mass transfer process. Post-adsorption characterization through XRD, FTIR, SEM-EDX, and zeta potential analyses confirmed partial MgO hydroxylation to Mg(OH)_2_, preservation of dye molecular structure, and multi-mechanism interactions involving electrostatic attraction, hydrogen bonding, and van der Waals forces. The adsorbent maintained robust performance across a practical pH range (7–9), exhibited minimal metal leaching under worst-case scenarios (all dissolved metals below USEPA/WHO/Egyptian limits), and demonstrated remarkable regenerability through acid-thermal treatment, retaining 78.5% capacity after 15 consecutive cycles—far exceeding the typical 5-cycle benchmark for industrial feasibility. Initial techno-economic analysis predicted a treatment cost of about 1.09 USD per cubic meter (m^3^) of water treated for a model 1000 m^3^ per day (d^−1^) plant that offered a cost saving of 90–95% compared to industrial adsorbents. These estimates need to be confirmed for a continuous flow process. This study offers proof of concept of DCI foundry waste as a useful absorbent for removing cationic dye in a laboratory setting. In future studies, it will be important to examine the potential for using this waste material to remove dyes that differ in structure namely, anionic, reactive, and disperse dyes from actual textile wastewater streams.

## Conflicts of interest

The authors declare that they have no known competing financial interests or personal relationships that could have appeared to influence the work reported in this paper.

## Supplementary Material

RA-016-D6RA02129H-s001

## Data Availability

The data supporting the findings of this study are available within the article and its supplementary information (SI). Supplementary information is available. See DOI: https://doi.org/10.1039/d6ra02129h.
