# Supplementary material for: Valorization of ductile cast iron solid waste as a high-performance adsorbent for crystal violet removal: characterization, optimization, and mechanistic insights
Source: RSC Adv. 2026 May 11;16(27):24427–53. doi: 10.1039/d6ra02129h (PMC13158850; doi:10.1039/d6ra02129h)
Supplement: RA-016-D6RA02129H-s001 [file RA-016-D6RA02129H-s001.pdf]

Supplementary Information (SI) for RSC Advances.
This journal is © The Royal Society of Chemistry 2026

Valorization of Ductile Cast Iron Solid Waste as a High-Performance Adsorbent for Crystal Violet Removal: Characterization, Optimization, and Mechanistic Insights

Ibrahim M. Ibrahim[a,b], A. M. Turky[b], Nasser Y. Mostafa[b], Mai H. Roushdy[a*]

Section S1. Detailed BET N_2 Adsorption-Desorption Isotherm Analysis

According to the IUPAC classification, The N_2 adsorption desorption isotherm (Figure 2a) exhibits Type IV behavior, which is a hallmark of mesoporous materials and is accompanied by different adsorption features in the various regions of relative pressure: I. This gradual increase at medium pressure-range (P/P_0 = 0.1 0.8) is attributed to the monolayer multilayer adsorption on mesopore walls, therefore meso-porosity is the main feature confirmed. II A significant nitrogen uptake happens at higher relative pressures (P/P_0 = 0.4 0.9) and this is a result of micropore filling which accounts for approximately 20% of the total pore volume and suggests that there are small pores (<2 nm) present, III The strong nitrogen uptake that is accompanied with an H3-type hysteresis loop is a clear indication of mesopores capillary condensation (2-50 nm), where the steep desorption branch and gradually sloping adsorption branch are characteristics of slit-shaped pores or aggregates of plate-like particles with irregular pore sizes.

BJH pore size distribution analysis (Figure 2b) of the desorption branch confirms the presence of a multimodal pore size distribution with clearly separated mesopore populations. A main mesopore peak at 3.5 4.2 nm constitutes approximately 60% of the total pore volume. It is thus a highly developed mesoporous structure that is perfectly adapted in terms of efficient accessibility of crystal violet molecules; secondary mesopores are the result of interparticle voids between aggregated nanopores; and a broad distribution tail of up to 30 40 nm at the end, which comprises only about 15% of the pore volume, is a sign of structural heterogeneity and macropore entrances.

Section S2. Stage-by-Stage TGA-DTG Analysis and Thermal Treatment Rationale

Thermogravimetric analysis coupled with derivative thermogravimetry (Figure 7) characterized thermal stability, decomposition behavior, and optimal regeneration temperature selection. TGA curve (weight % vs. temperature, 25-900°C, 10°C/min heating rate, air atmosphere) shows the total weight loss is 8.0% over 25-900°C range, indicating high thermal stability. Stage 1 (25-150°C) includes 2.8% weight loss with DTG endothermic peak at 85°C which happened due to Physisorbed water evaporation (surface moisture, capillary water in mesopores) and monolayer water coverage on MgO surface, reasonable for material stored at ambient humidity. Stage 2 (150-350°C) include 3.5% weight loss with DTG endothermic peak at 285°C which happened due to surface dehydroxylation (2 Mg-OH → Mg-O-Mg + H_2O) and decomposition of residual organic contaminants (Organic residues → CO_2 + H_2O). Stage 3 (350-550°C) which includes1.2% weight loss with weak DTG peak at 420°C due to further dehydroxylation of more stable Mg-OH groups (interior layers, strongly bound) and structural water removal from hydrated phases as relatively small loss indicates most volatile/labile species removed by 350°C, validating 400°C activation and 300°C regeneration temperatures [85]. Stage 4 (550-900°C) with changes <0.5% and no significant DTG peaks because of Minimal change happened to MgO, ZnO, Fe_2O_3 phases that confirms material purity and safety (no spontaneous combustion risk). No phase transitions because of the absence of sharp endo/exothermic events at characteristic temperatures confirms no polymorphic transitions (MgO cubic structure stable), no melting, no decomposition of major phases.

Based on the thermal analysis data the thermal activation (400°C, 3 h) as this temperature removes physisorbed water (Stage 1) and surface hydroxyl groups (Stage 2), creating coordinatively unsaturated Mg^{2+} sites ideal for adsorption. The regeneration temperature (300°C, 2 h) within Stage 2 region, sufficient to decompose adsorbed organic dye (CV expected decomposition 200-350°C based on TGA of pure CV, not shown) while avoiding excessive dehydroxylation that would require rehydration. Post-regeneration, material spontaneously rehydroxylates

upon ambient air exposure, restoring active sites. Above 450-500°C (Stage 3 completion), sintering risk increases. Particle growth and surface area loss observed when heated >600°C (surface area drops to 198 m²/g at 600°C, 162 m²/g at 800°C in separate experiments). Therefore, all thermal treatments maintained ≤400°C. DCI waste withstands multiple regeneration cycles (15 tested) at 300°C without structural degradation, contrasting with some organic adsorbents (activated carbon oxidizes in air >400°C, some biochar unstable >350°C). No exothermic decomposition, no rapid gas evolution (DTG slopes gradual), indicates safe handling and processing. Material non-flammable, non-explosive under normal conditions.

Table S1. Kinetic Model Statistical Comparison

Table S1: Comparison of Kinetic Models for the Adsorption of CV onto DCI Waste (C_0 = 40 mg/L, Dose = 6.2 g/L, pH = 8.0, T = 25 °C). q_e,exp = 0.121 mg/g (Equilibrium capacity from experiment). Determined by non-linear regression. Percentage error of q_e = |q_e,calc – q_e,exp| / q_e.

**Kinetic Model**	**R^2**	**q_e,calc (mg g^{-1})**	**q_e,exp (mg g^{-1})**	**q_e error (%)**	**Assessment**
Pseudo-first-order	0.9870	0.118	0.121	2.5	✓ Best fit — excellent R^2 and <3% q_e error
Pseudo-second-order	0.8499	0.087	0.121	28.1	✗ Poor fit — large q_e discrepancy (28%)
Elovich	NR	NR	0.121	NR	Chemisorption-surface model; qualitative only
Weber–Morris (IPD)	Stage-dependent: Stage 1: 0.994 Stage 2: 0.989 Stage 3: 0.982	NR	0.121	NR	Multi-stage; not a single-equation fit
Boyd	NR	NR	0.121	NR	Mechanistic diffusion model; not regression-based

Key Points: NR = not relevant; The Weber-Morris (IPD) and Boyd models are mechanistic multiple-stage models, which cannot be used to determine a single regression coefficient R^2 or q_e value. The pseudo-first-order kinetic model is validated due to its high R^2 value (0.9870) and minimum q_e error percentage (2.5%). Therefore, the adsorption process is physisorption. The pseudo-second-order kinetic model is invalid due to its low R^2 value (0.8499) and high q_e error percentage (28.1%). Thus, the process does not involve chemisorption.
